# Mosquito Exosomal Tetraspanin CD151 Facilitates Flaviviral Transmission and Interacts with ZIKV and DENV2 Viral Proteins

**DOI:** 10.3390/ijms26157394

**Published:** 2025-07-31

**Authors:** Durga Neupane, Md Bayzid, Girish Neelakanta, Hameeda Sultana

**Affiliations:** Department of Biomedical and Diagnostic Sciences, College of Veterinary Medicine, University of Tennessee, Knoxville, TN 37996, USA

**Keywords:** mosquito cells, ZIKA virus, dengue virus, tetraspanins, CD151, EVs, GW4869

## Abstract

The expanding distribution and geographic range of mosquitoes have potentially contributed to increased flaviviral dissemination and transmission. Despite the growing burden of flaviviral infections, there are no effective antiviral treatments or vaccines, highlighting the need for novel therapeutic targets. Tetraspanins, a superfamily of transmembrane domain glycoproteins involved in cellular organization, signaling, and protein–protein interactions have been recognized as potential mediators of flaviviral infection and transmission. While their roles in vertebrate hosts have been explored, their involvement in flaviviral replication and dissemination within medically important vectors remains poorly understood. In this study, we investigated the role of arthropod tetraspanins in mosquito cells and extracellular vesicles (EVs) derived from cells infected with Zika virus (ZIKV) and dengue virus (serotype 2; DENV2). Among several of the tetraspanins analyzed, only CD151 was significantly upregulated in both mosquito cells and in EVs derived from ZIKV/DENV2-infected cells. RNAi-mediated silencing of CD151 led to a marked reduction in viral burden, suggesting its crucial role in flavivirus replication. Inhibition of EV biogenesis using GW4869 further demonstrated that EV-mediated viral transmission contributes to flavivirus propagation. Additionally, co-immunoprecipitation and immunofluorescence analyses revealed direct interactions between CD151 and ZIKV NS2B and DENV2 capsid proteins. Overall, our findings highlight the functional importance of mosquito CD151 in the replication and transmission of ZIKV and DENV2. This study provides new insights into the molecular mechanisms of flaviviral infection in mosquitoes and suggests that targeting vector tetraspanins may offer a potential approach to controlling mosquito-borne flaviviruses.

## 1. Introduction

Mosquito-borne flaviviruses include clinically important human pathogens such as Zika virus (ZIKV), dengue virus (DENV), West Nile virus (WNV), and St. Louis encephalitis virus (SLEV) that cause a wide range of symptoms such as fever, congenital anomalies, hemorrhagic syndromes, multiple organ failures, paralysis, meningitis, encephalitis and ultimately death [1,2,3,4]. Flaviviruses are widely distributed and transmitted to humans through the bite of an arthropod vector such as an infected mosquito. Bites from infected mosquitoes result in substantial morbidity, mortality, significant health concerns, and socioeconomic burden [3,5]. However, to this date, there are no effective vaccines or treatments available for pan-flavivirus infections. There are two approved vaccines (Dengvaxia or Qdenga from Sanofi or Takeda) that are available to treat DENV infections in humans [6,7,8]. Therefore, understanding the molecular mechanisms underlying their infection and pathogenesis in humans and mosquitoes is important for the development of effective therapeutic interventions and vaccines [1,2,4,5,9,10,11]. Our previous study showed that mosquito tetraspanin-domain-containing glycoprotein Tsp29Fb (an ortholog of human CD63) facilitates transmission of DENV via exosomes. We found that DENV2/3 (serotypes 2 or 3) RNA, the full-length viral RNA genome (of DENV2), and viral proteins/polyproteins were transmitted via exosomes/extracellular vesicles (EVs) from mosquito donor cells to naïve recipient mammalian cells [12,13]. Tetraspanins are a superfamily of four-transmembrane-domain-containing glycoproteins that carry a specific site for facilitating protein–protein interaction(s) [14,15,16,17]. The hydrophobic transmembrane domains enable the formation of tetraspanin-enriched microdomains (TEMs) [14,15,16,17]. Tetraspanins facilitate interactions with a diverse group of proteins such as integrins, metalloproteases, intracellular signaling molecules, immunoglobulin superfamily receptor(s), and other members of the tetraspanin family of proteins [14,15,16,17]. These interactions with other proteins suggest tetraspanins as important regulators for cellular functions, including cell migration, signal transduction, adhesion, intracellular trafficking, and EV biogenesis, in mammalian cells [13,14,15,16,17,18].

Recent research has reported the involvement of tetraspanins in facilitating viral entry/exit, in cell–cell spreading/dissemination, and in transmission from arthropod to mammalian cells [12,13,19,20,21,22,23]. Also, it has been shown that CD189 is involved in the intracellular spread of viral particles between mosquito cells and that mammalian tetraspanin CD81 acts as a receptor for Hepatitis C virus (HCV) to facilitate its entry into host cells [24,25,26,27]. The broad tissue distribution of tetraspanins and their crucial functions at the cell surface makes them potentially useful for pathogens in terms of receptor binding, entry, trafficking, replication, nuclear transport, virus assembly, budding, and exit [13,15,18,19,20,21,22,23,24,25,28,29,30,31]. Tetraspanins regulate receptor internalization process but also serve as reliable markers for exosomes, thus suggesting these proteins as crucial transporters of pathogens from infected cells to naïve recipient cells [13,14,18,19,20,21,22,23,24,25,28,29,30]. To date, very little is known about the putative role of tetraspanins and their involvement in exosomal cargo selection [13,22]. Tetraspanins have been reported to be highly expressed in EVs and are involved in various functions such as cell–cell communication, platelet activation, EV biogenesis, and viral infections [12,13,18,32,33]. A few studies have reported that viral infections could modify EV biogenesis and cargo sorting mechanisms [34,35,36,37,38,39]. CD151, an important member of tetraspanin superfamily, has shown to play a significant role in the viral penetration of human cytomegalovirus (CMV) and aids in the nuclear export signaling of human influenza virus, thus suggesting a novel role for this molecule in viral infections [28,40]. We hypothesized that tetraspanins regulate exosome biogenesis upon viral infection; however, their role is not yet explored in medically important vectors. The role of the specific tetraspanin CD151 in flavivirus infection is not yet addressed in arthropods. Hence, we investigated the role of mosquito CD151 (a human ortholog of CD151) in ZIKV/DENV2 infection to understand the molecular mechanism of CD151-mediated flavivirus infections.

## 2. Results

### 2.1. Bioinformatic and Prediction Analysis of Tetraspanins Combed from the Aedes aegypti Genome

Protein sequences of *Aedes aegypti* mosquito tetraspanins were collected from NCBI. Detailed information regarding the protein name/accession numbers, their respective nucleotide accession numbers, and VectorBase accession numbers along with signal peptide information is shown in Table S1. Amino acid sequence alignment of mosquito tetraspanins performed in DNASTAR (Lasergene) software (https://www.dnastar.com/software/lasergene, accessed on 28 April 2025) using ClustalW alignment program (MegAlign Pro 17.3) that showed some degree of conservation (Figure S1A). Conserved domain sequences of different mosquito tetraspanins are shaded in black for identification (Figure S1A). The amino acid sequence alignment revealed 24–30% identity between different mosquito tetraspanins (Figure S1B). Phylogenetic analysis revealed that *A. aegypti* tetraspanins forms different clades (Figure S1C). The CD151 and TSP-11 amino acid sequences shared the clade and were close to TSP-9 and TSP-33, but they were far distant from TSP-2 and the others (Figure S1C). In addition, we noted that multiple sequence alignments performed with the mosquito tetraspanin CD151 amino acid sequence and other orthologs (*Aedes albopictus* mosquito, *Ixodes scapularis* tick, *Drosophila melanogaster* fly, mouse-*Mus musculus* and human-*Homo sapiens*) revealed conserved residues (that are shown as shades) and some degree of conservation (Figure S2A). The amino acid sequence alignment of mosquito CD151 with other orthologs revealed 98% identity with *A. albopictus* protein, 44–45% identity with human, mouse, and *I. scapularis* tick proteins, and 26% identity with *D. melanogaster* protein (Figure S2B). Phylogenetic analysis further showed that *A. aegypti* CD151 fell within the same clade as the *A. albopictus* ortholog (Figure S2C). The mouse and human CD151 sequences fall within the same clade, which is distant from the mosquito CD151 sequence clade (Figure S2C). We also analyzed the number of amino acids (Figure S3A), number of transmembrane (TM) domains (Figure S3B) and post-translational modification sites such as ASN glycosylation (Figure S3C), number of myristoylation sites (Figure S3D), protein kinase C (PKC) phosphorylation sites (Figure S3E), and number of CK2 phosphorylation sites (Figure S3F) using PROSITE. The bioinformatics data obtained from *A. aegypti* mosquito tetraspanins showed that they were of sizes 200–300 amino acids and all had the presence of four transmembrane domains and contained several protein modification sites, as revealed by the detailed prediction analysis (Figure S3).

### 2.2. ZIKV and DENV2 Infection Selectively Modulates Tetraspanin cd151 Gene Expression in Mosquito Cells and Derived EVs

**Figure 1:**
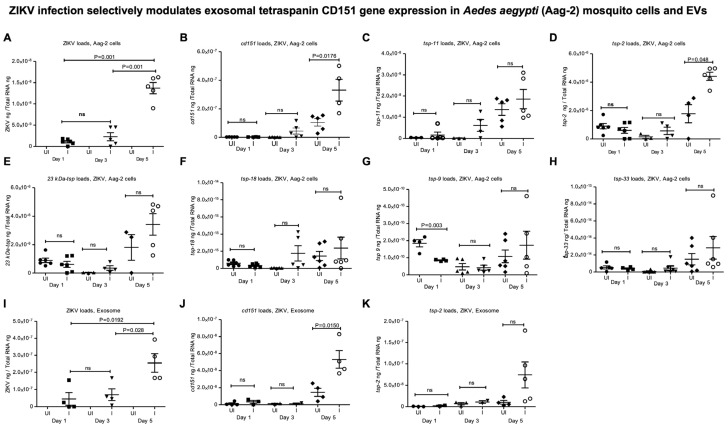
ZIKV infection selectively modulates mosquito tetraspanin gene expression in *Aedes aegypti* (Aag-2) mosquito cells and upregulates mosquito tetraspanin *cd151* gene expression alone in Aag-2-derived EVs. QRT-PCR analysis showing viral burden (determined as ZIKV NS5 transcript levels) (**A**) or gene expression of *cd151* (**B**), *tsp-11* (**C**), *tsp-2* (**D**), *23 kDa tsp protein* (**E**), *tsp-18* (**F**), *tsp-9* (**G**), and *tsp-33* (**H**) in Aag-2 cells infected (**I**) with 5 MOI of ZIKV and tested at different time points (days 1, 3, and 5 post-infection, p.i.). QRT-PCR analysis showing viral loads (**I**) or gene expression of *cd151* (**J**) and *tsp-2* (**K**) in EVs derived from Aag-2 cells infected (**I**) with ZIKV (5 MOI) and tested at different time points (days 1, 3, and 5 p.i.). Uninfected (UI) Aag-2 cells or exosomes derived from UI Aag-2 cells served as controls in all expression analyses. In each panel, transcript levels were normalized to total RNA levels. Each data point represents one independent culture well in a plate and each group had four–six independent replicates. Statistical significance was determined by unpaired two-tailed *t* test and ns indicates not significant. The *p* values that are less than 0.05 are considered as significant.

**Figure 2:**
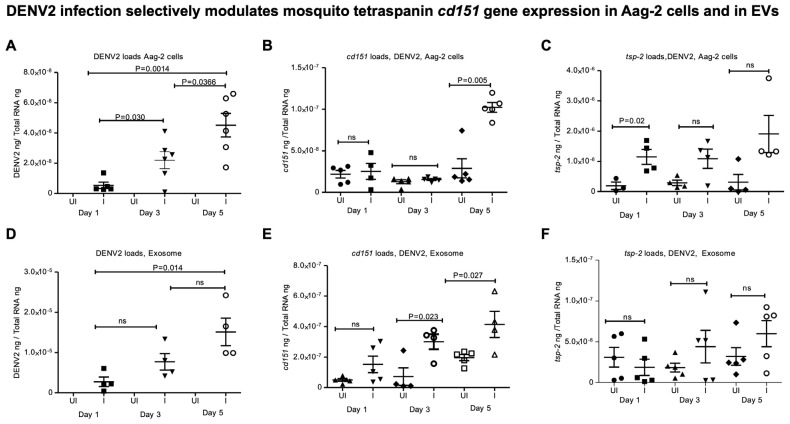
DENV2 infection selectively upregulates mosquito tetraspanin *cd151* gene expression in Aag-2 cells and derived EVs. QRT-PCR analysis showing DENV2 viral burden (**A**,**D**) (determined as capsid transcripts) or *cd151* (**B**,**E**) and *tsp-2* (**C**,**F**) gene expression in Aag-2 cells (**A**–**C**) and in EVs derived from Aag-2 cells (**D**–**F**) infected (I) with 5 MOI of DENV2 and tested at different time points (days 1, 3, and 5 p.i.), respectively. Uninfected (UI) cells serve as controls in all panels. In each panel, transcript levels were normalized to total RNA levels. Each data point represents one independent culture well in a plate and each treatment group had four–six independent replicates. Statistical significance was determined by unpaired two-tailed *t* test and ns indicates not significant. The *p* values that are less than 0.05 are considered as significant.

First, we amplified the mosquito tetraspanin (*tsp*) gene (*cd151*, *tsp*-11, *tsp*-2, *23-kDa tsp*, *tsp*-18, *tsp*-9, and *tsp*-33) fragments (of ~ 200 bps) from *A. aegypti* (Aag-2 cells) in vitro cell line (Figure S4A) and the oligonucleotides are shown in Table S2. To understand whether ZIKV infection has any impact on the expression of these tetraspanin genes, we infected Aag-2 cells with ZIKV (at 5 MOI) and collected samples at different time points (days 1, 3, and 5 post-infection, p.i.) (Figure 1). QRT-PCR analysis showed significantly (*p* < 0.05) increasing ZIKV loads over the time course of infection from days 1, 3, and 5 p.i. (Figure 1A). ZIKV loads were significantly higher in Aag2 cells at day 5 p.i. when compared to the viral loads noted at days 1 and 3 p.i. (Figure 1A). Expression of *cd151* (Figure 1B) and *tsp-2* (Figure 1D) was noted to be significantly upregulated at day 5 p.i. in ZIKV-infected Aag2 cells compared to the levels noted in uninfected controls. The expression of *tsp-9* was noted to be significantly downregulated at day 1 p.i. in ZIKV-infected Aag2 cells compared to the levels noted in uninfected controls (Figure 1G). However, no significant differences were noted in the expression levels of *tsp-11* (Figure 1C), *23-kDa-tsp* (Figure 1E), *tsp-18* (Figure 1F), and *tsp-33* (Figure 1H) between uninfected or ZIKV-infected Aag2 cells (at all tested time points). Furthermore, viral loads (viral RNA genomes/viral RNA and proteins/polyproteins that are packaged within the mosquito-cell-derived EVs) were also noted to be higher in EVs derived from ZIKV-infected Aag-2 cells at day 5 p.i. compared to the loads noted at earlier time points of days 1 and 3 p.i. (Figure 1I). In addition, significant upregulation of *cd151* was also noted in EVs derived from ZIKV-infected Aag-2 cells when compared to the levels noted in EVs derived from uninfected cells at day 5 p.i. (Figure 1J). No significant differences were noted in *tsp-2* expression in EVs at any tested time points of ZIKV infection (Figure 1K). QRT-PCR analysis further showed that viral loads in Aag-2 cells increased significantly (*p* < 0.05) over the time course of days 1, 3, and 5 after DENV2 infection (Figure 2A). We also observed similar significant upregulation of *cd151* gene expression in DENV2-infected Aag-2 cells in comparison to the expression noted in the uninfected control group at day 5 p.i. (Figure 2B). Gene expression of *tsp-2* showed significant upregulation only at day 1 post-DENV2-infection in Aag-2 cells but not at later time points (of days 3 and 5) in comparison to their respective uninfected controls (Figure 2C). QRT-PCR analysis with EVs derived from DENV2-infected Aag-2 cells showed that viral loads were significantly (*p* < 0.05) higher at day 5 p.i. in comparison to the loads noted at the other two time points (Figure 2D). Significantly increased *cd151* transcript levels were detected in EVs derived from DENV2-infected Aag-2 cells compared to the levels detected in EVs derived from uninfected controls at days 3 and 5 p.i but not at day 1 p.i. (Figure 2E). However, no significant differences were observed in the detection of *tsp-2* transcripts in EVs derived from DENV2-infected Aag2 cells in comparison to the uninfected controls (at all tested time points) (Figure 2F). Since no significant differences in the detection of *tsp-2* transcripts were noted in EVs derived from DENV2-infected Aag2 cells and uninfected controls, we considered investigating only exosomal *cd151* for further analysis.

**Figure 3:**
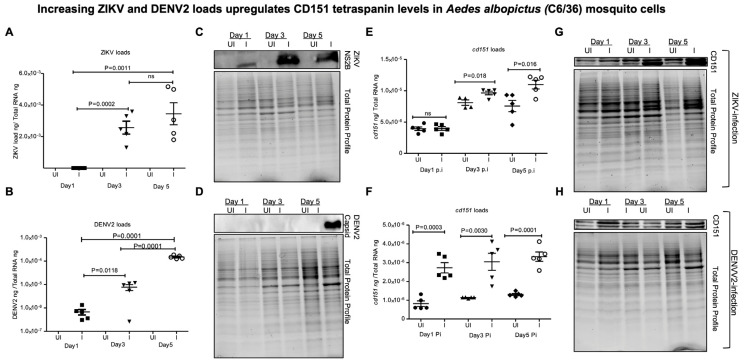
Increasing ZIKV and DENV2 loads upregulate CD151 levels in *Aedes albopictus* (C6/36) mosquito cells. QRT-PCR analysis showing ZIKV (**A**) or DENV2 (**B**) loads in C6/36 cells infected (I) with 5 MOI (of each virus) and tested at time points of days 1, 3 and 5 p.i. Immunoblotting analysis showing ZIKV NS2B protein (**C**) or DENV2 capsid protein (**D**) levels in C6/36 cells infected with ZIKV or DENV2 (with 5 MOI of each virus) and at tested time points of days 1, 3 and 5 p.i. QRT-PCR analysis showing *cd151* transcript levels in ZIKV (**E**) or DENV2 (**F**) loads in C6/36 cells (with 5 MOI of each) and at tested time points of days 1, 3, and 5 p.i. Immunoblotting analysis showing CD151 protein loads in ZIKV- (**G**) or in DENV2- (**H**) infected C6/36 cells (infected with 5 MOI of each virus) and at tested time points of days 1, 3, and 5 p.i. Transcript levels were normalized to total RNA levels. Each data point represents one independent culture well in a plate and each treatment group had five independent replicates. Statistical significance was determined by unpaired two-tailed *t* test and ns indicates not significant. The *p* values that are less than 0.05 are considered as significant. In immunoblots, the total protein profile gel images serve as loading controls.

To examine whether ZIKV/DENV2 infection also modulates *cd151* gene expression in *A. albopictus* cells, we infected the C6/36 cell line and collected both cells and EVs at days 1, 3, and 5 post-ZIKV/DENV2-infection. QRT-PCR analysis showed significantly increasing ZIKV (Figure 3A) and DENV2 (Figure 3B) loads at days 3 and 5 p.i. when compared to the loads noted at day 1 p.i.. These results were further supported by immunoblotting analysis performed on total cell lysates collected from ZIKV/DENV2 infection kinetics on days 1, 3, and 5 p.i. (Figure 3C,D). We noted dramatic increase in ZIKV NS2B protein loads at all tested time points of days 1, 3, and 5 p.i., whereas DENV2 capsid protein was found to be enhanced at day 5 p.i. (Figure 3C,D). ZIKV NS2B protein showed increasing loads at day 3, and we also noted it in a modified form with increasing intensity (Figure 3C). Protein profile gel images of total cell lysates served as loading controls (Figure 3C,D). We also observed significantly increased *cd151* transcript levels in ZIKV-infected C6/36 cells when compared to the levels noted in uninfected controls at days 3 and 5 p.i. (Figure 3E). No differences were noted at day 1 post-ZIKV-infection (Figure 3E). The *cd151* transcript levels were significantly upregulated at all tested time points of days 1, 3, and 5 post-DENV2-infection in C6/36 cells (Figure 3F). Immunoblotting analysis correlated with QRT-PCR data, where dramatically increased CD151 protein levels were evident upon ZIKV (Figure 3G) or DENV2 (Figure 3H) infection in C6/36 cells when compared to the levels noted in their respective uninfected controls (Figure 3G,H). CD151 protein was noted as a doublet, suggesting induction in its modified form (may be palmitoylation) (Figure 3G,H). Full-length immunoblots and total protein profile gel images for CD151 expression upon ZIKV and DENV2 infection are shown (Figure S4B–E). These data show that ZIKV and DENV2 selectively upregulate CD151 levels in both Aag-2 and C6/36 cells.

### 2.3. RNAi-Mediated Silencing of cd151 Reduces ZIKV and DENV2 Burden in Aag-2 Cells

**Figure 4:**
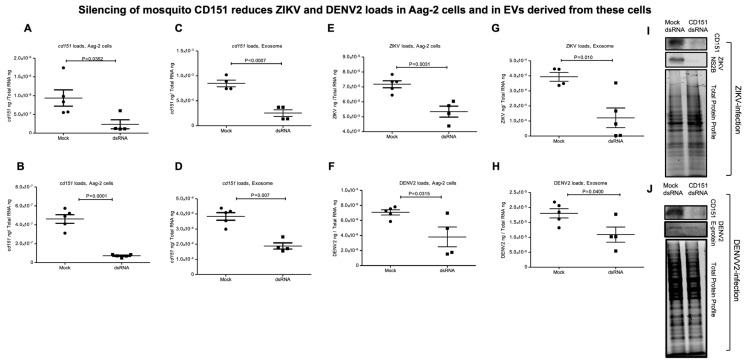
Silencing of mosquito CD151 reduces ZIKV and DENV2 loads in Aag-2 cells and in EVs derived from these cells. QRT-PCR analysis showing expression of *cd151* to reveal the silencing efficiency in ZIKV-infected (at 5 MOI) (**A**,**C**) or DENV2-infected (at 2 MOI) (**B**,**D**) Aag-2 cells (**A**,**B**) or in EVs derived from these Aag-2 cells (**C**,**D**), respectively. Viral loads in Aag-2 cells (**E**,**F**) infected with ZIKV (**E**) or DENV2 (**F**) and in EVs (**G**,**H**) derived from ZIKV-infected (**G**) or DENV2-infected (**H**) Aag-2 cells treated with mock or *cd151* dsRNA for 48 h post-transfection or 24 h p.i. are shown. Transcript levels of *cd151*, DENV2, and ZIKV RNA levels were all normalized to total RNA levels. Each data point represents one independent culture well in a plate and each treatment group had four–five independent replicates. Statistical significance was determined using unpaired two-tailed *t* tests. The *p* values that are less than 0.05 are considered as significant. Panels (**I**) and (**J**) show the immunoblotting analysis for detection of CD151 to reveal the silencing efficiency and ZIKV-NS2B or DENV2 capsid proteins to show viral loads in Aag-2 cells treated with mock or *cd151* dsRNAs. Total protein profile gel images serve as loading controls.

**Figure 5:**
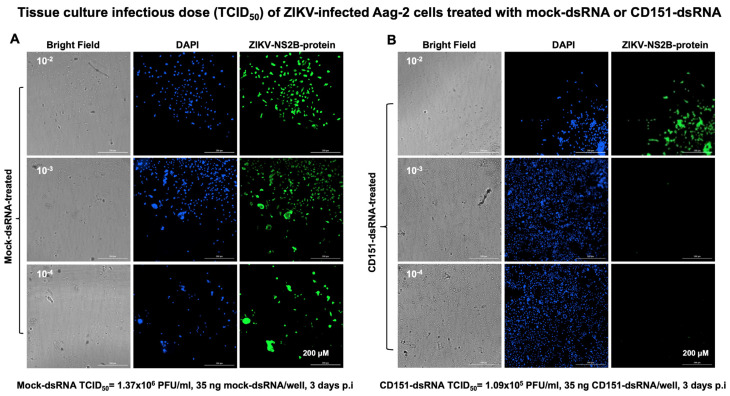
ZIKV infectivity is reduced upon CD151 silencing in Aag-2 cells. Viral dilution or infectivity assay showing the tissue culture infectious dose (TCID_50_) that determines the viral infectivity (by immunofluorescence assay) in mock-dsRNA-treated (**A**), or *cd151*-dsRNA-treated (**B**) Aag-2 cells infected with ZIKV (for 3 days p.i.) and at different dilutions of 10^−2^, 10^−3^, and 10^−4^. Bright field images of Aag-2 cells from ZIKV-infected groups are shown in the left column. DAPI-stained nuclei images are shown in blue (and in the middle column). ZIKV-NS2B viral protein staining is shown in green (in the right column). The TCID_50_ dose is indicated at the bottom of each panel. Images are obtained at 20× magnification. Scale bar indicates 200 µM in each image and is highlighted in the last image of the panel.

**Figure 6:**
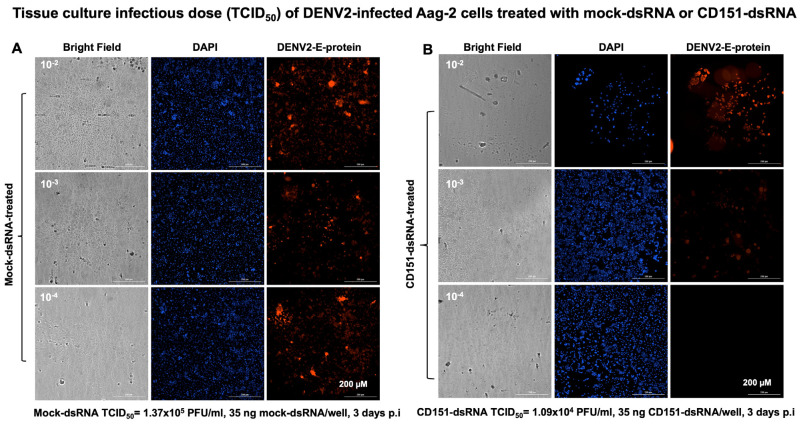
DENV2 infectivity is reduced upon CD151 silencing in Aag-2 cells. Viral dilution or infectivity assay showing the tissue culture infectious dose (TCID_50_) that determines the viral infectivity (by immunofluorescence assay) in mock-dsRNA-treated (**A**), or *cd151*-dsRNA-treated (**B**) Aag-2 cells infected with DENV2 (for 3 days p.i.) and at different dilutions of 10^−2^, 10^−3^, and 10^−4^. Bright field images of Aag-2 cells from DENV2-infected groups are shown in the left column. DAPI-stained nuclei images are shown in blue (and in the middle column). DENV2 capsid viral protein staining is shown in green (and in the right column). TCID_50_ dose is indicated at the bottom of each panel. Images are obtained at 20× magnification. Scale bar indicates 200 µM in each image and is highlighted in the last image of the panel.

To understand the role of CD151 in ZIKV/DENV2 infection, we employed the RNA interference method. We first amplified the *A. aegypti* mosquito *cd151* gene fragment (of size 285 bp) (Figure S5A) and cloned it into the dsRNA vector pL4440 (as described in methods) (Figure S5B). Clones were screened by colony PCR (Figure S5C) and dsRNA was synthesized from selected clones (as shown with arrows in Figure S5C) and collected as three elutions (Figure S5D). Aag-2 cells (1 × 10^6^ cells/well) were transfected with mock (empty pL4440 plasmid) or *cd151* dsRNA (750 ng/well for each) and lipofectamine for 48 h followed by ZIKV/DENV2 infection (5 MOI/2 MOI) for an additional 24 h. Bright field images collected as before transfection and at 4, 24, and 48 h post-transfection or 24 h post-ZIKV/DENV2-infection are shown to assess morphological changes (Figure S6). QRT-PCR analysis showed significantly (*p* < 0.05) decreased *cd151* transcript levels in *cd151*-dsRNA-treated ZIKV- (Figure 4A) or DENV2-infected cells (Figure 4B) when compared to the levels noted in ZIKV/DENV2-infected mock-dsRNA-treated control groups (Figure 4A,B). In addition, we also noted that the silencing of *cd151* in Aag-2 cells resulted in significantly (*p* < 0.05) decreased *cd151* transcripts in EVs derived from *cd151*-dsRNA-treated ZIKV- (Figure 4C) or DENV2-infected Aag-2 cells (Figure 4D) in comparison to their respective mock controls (Figure 4C,D). Both ZIKV/DENV2 loads were also significantly reduced in *cd151*-dsRNA-treated ZIKV- (Figure 4E) or DENV2-infected Aag-2 cells (Figure 4F) and in EVs derived from these silenced cells (Figure 4G,H). Furthermore, immunoblotting analysis showed similar results, with reduced CD151 protein levels in *cd151*-dsRNA-treated ZIKV- (Figure 4I) or DENV2-infected Aag-2 cells (Figure 4J) when compared to their respective mock-dsRNA-treated control groups (Figure 4I,J). Full-length immunoblots for CD151 protein expression in *cd151*-dsRNA or mock-dsRNA-treated and ZIKV- or DENV2-infected groups are shown (Figure S5E). The total protein profile gel image for these silenced samples serves as a loading control (Figure S5F). We also observed dramatically reduced ZIKV (NS2B)/DENV2 E-protein levels in Aag-2 cell lysates (Figure 4I,J) from these silenced cells (Figure 4I,J). To further address the effects of CD151 silencing, we included the viral yield data by performing viral dilution/infectivity assays to reveal the production of infectious viral particles. We noted that *cd151*-dsRNA-treated (transfected with 35 ng/well of a 96 well plate, for 24 h) and ZIKV-infected (for additional 3 days p.i.) Aag-2 cells showed reduced viral infectivity (as revealed by the viral dilution assay) in comparison to the ZIKV-infected-mock-dsRNA-treated control group (Figure 5A,B). A tissue culture infectious dose (TCID_50_) of 1.09 × 10^5^ pfu/mL was determined for the *cd151*-dsRNA-treated group; the mock-dsRNA-treated group had a TCID_50_ value of 1.37 × 10^6^ pfu/mL. A similar experiment in the context of DENV2 infection indicated a TCID_50_ value of 1.09 × 10^4^ pfu/mL for the *cd151*-dsRNA-treated group in comparison to the mock-dsRNA-treated group, which showed a TCID_50_ value of 1.37 × 10^5^ pfu/mL (Figure 6A,B). We observed that infection was reduced by a log in DENV2 in comparison to ZIKV infection and for mock/*cd151*-dsRNA treated groups, respectively (Figure 5 and Figure 6). These results indicate that silencing of *cd151* expression affects both the ZIKV and DENV2 burden in mosquito cells.

### 2.4. CD151 Antibody or GW4869 Treatment Reduces ZIKV and DENV2 Burden in Mosquito Cells

**Figure 7:**
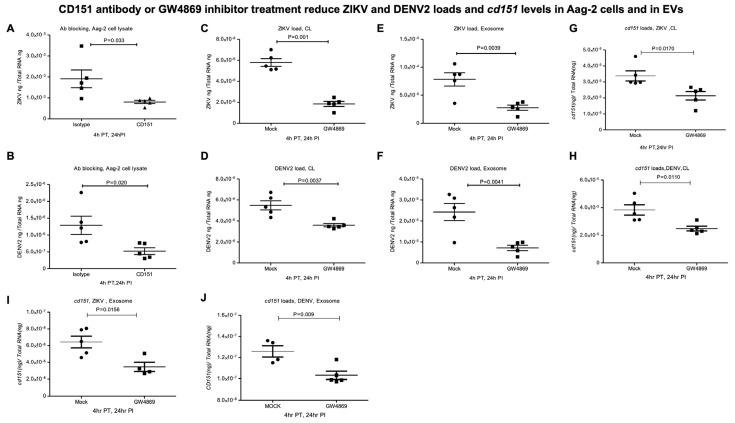
CD151 antibody blocking and GW4869 treatment affects ZIKV and DENV2 loads in Aag-2 cells. QRT-PCR analysis showing viral loads in Aag-2 cells treated (for 4 h) with 2 µg of CD151 or isotype control rabbit polyclonal antibodies followed by infection (at 5 MOI) with ZIKV (**A**) or DENV2 (**B**) for 24 h. QRT-PCR analysis showing ZIKV (**C**,**E**) or DENV2 loads (**D**,**F**) and *cd151* transcripts (**G**–**J**) in Aag-2 cells (**C**,**D**,**G**,**H**) treated with 10 µM GW4869 inhibitor (for 4 h) or mock control (0.1% DMSO) followed by viral infection (5 MOI) for 24 h post-infection and in derived EVs (**E**,**F**,**I**,**J**). DMSO was used as a control for these inhibitor experiments. Transcript levels of *cd151*, DENV2, and ZIKV RNA levels were all normalized to total RNA levels. Each data point represents one independent culture well in a plate and each treatment group had five independent replicates. Statistical significance was determined using unpaired two-tailed *t* test. The *p* values that are less than 0.05 are considered as significant.

First, we tested the effects of functional antibody blocking with a highly cross-reactive human CD151 antibody in Aag-2 mosquito cells. QRT-PCR analysis showed that Aag-2 cells treated with only 2 µg of CD151 antibody (for 4 h) followed by ZIKV/DENV2 infection (at 5 MOI, for additional 24 h) significantly reduced the viral loads (Figure 7A,B) in comparison to the isotype control antibody treatment. Furthermore, we addressed the production of infectious viral particles or yield by performing virus dilution assays. We noted that CD151-antibody-treated (with 0.3 µg/well of a 96 well plate, for 4 h) and ZIKV-infected (for 3 days p.i., viral dilution assay revealing the infectivity) Aag-2 cells showed reduced viral infectivity in comparison to the isotype-antibody-treated control group (Figure S7A,B). A TCID_50_ value of 7.37 × 10^4^ pfu/mL was determined for CD151-antibody-treated group; the isotype-antibody-treated control group showed a TCID_50_ value of 1.37 × 10^5^ pfu/mL. A similar experiment but using DENV2 infection indicated a TCID_50_ value of 6.13 × 10^4^ pfu/mL for the CD151-antibody-treated group, while the isotype-antibody-treated control group showed a TCID_50_ value of 1.09 × 10^5^ pfu/mL (Figure S8A,B).

**Figure 8:**
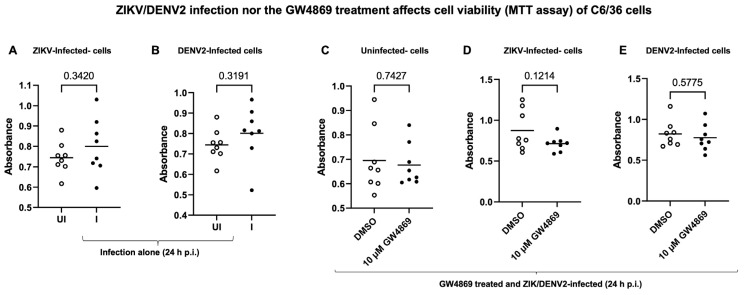
MTT assays revealed that neither ZIKV/DENV2 infection nor GW4869 treatment affects the viability of C6/36 cells. C6/36 cell viability measured by MTT assays performed in ZIKV-infected (**A**) or DENV2-infceted (**B**) groups at 24 h p.i. is shown. Uninfected (UI) cells serve as controls. MTT assays performed with uninfected C6/36 cells (**C**) treated with mock (0.1% DMSO) or GW4869 (10 µM) for 4 h and incubated for additional 24 h p.i. are shown. Cell viability measurement upon mock/GW4869 treatments (as above) and followed by infection (for 24 h p.i.) with ZIKV (**D**) or DENV2 (**E**) infection in C6/36 cells is shown. The mock-treated group serves as a control. In each panel, cell viability is determined independently. Each data point represents one independent culture well in a 96-well plate and each treatment group had eight independent replicates. Statistical significance was determined by unpaired two-tailed *t* test with Welch’s correction; *p* values that are less than 0.05 are considered as significant.

Next, we tested the effects of GW4869, a cell-permeable selective inhibitor for the enzyme neutral sphingomyelinase (nSMase2/SMPD3), which is required for exosome production and release. Bright field images collected as before GW4869 treatment or after treatment (at 4 or 28 h post-treatment or 24 h post-ZIKV/DENV2-infection) showed no morphological changes in Aag-2 cells treated (at 10 µM, for 4 h) with GW4869 inhibitor (Figure S9). QRT-PCR analysis showed that treatment of Aag-2 cells with GW4869 (at 10 µM, for 4 h) followed by ZIKV/DENV2 infection (at 5 MOI for an additional 24 h after infection) resulted in a significant reduction in viral burden in comparison to their respective mock (DMSO) control groups (Figure 7C,D). Also, we noted a significant reduction in ZIKV/DENV2 viral burden in EVs derived from Aag-2 cells treated with GW4869 inhibitor (Figure 7E,F). Furthermore, viral infectivity or the production of infectious viral particles, as determined by viral dilution assay, revealed that C6/36 cells treated with GW4869 (10 µM for 4 h) or with a mock control (0.1% DMSO, 4 h) followed by ZIKV infection (for 3 days p.i.) showed reduced viral infectivity in comparison to the mock-treated control group (Figure S10A,B). A TCID_50_ value of 2.34 × 10^4^ pfu/mL was determined for the GW4869-treated group, in comparison to the mock-treated group that showed a TCID_50_ of 1.09 × 10^5^ pfu/mL. A similar experiment with DENV2 infection indicated a tissue culture infectious dose (TCID_50_) of 6.14 × 10^4^ pfu/mL for the GW4869-treated group, in comparison to the mock-treated group that showed a TCID_50_ of 2.34 × 10^5^ pfu/mL (Figure S11A,B). In addition, we noted a significant reduction in *cd151* transcript levels in the GW4869-treated ZIKV (Figure 7G) or DENV2-infected Aag-2 cells (Figure 7H) and in EVs derived from these cells (Figure 7I,J) when compared to the levels noted in their respective mock-treated control cells or EVs. To understand the reduction in *cd151* transcript levels upon GW4869 treatment and upon ZIKV/DENV2 infections (shown in Figure 7G–J), we performed MTT assays, which revealed no significant differences in the viability of C6/36 cells infected with ZIKV (Figure 8A) or DENV2 (Figure 8B). We also noted no significant differences in the viability of C6/36 cells treated with GW4869 (10 µM for 4 h) or mock treated (0.1% DMSO, 4 h) group followed by ZIKV/DENV2 infection (for 3 days p.i.) or an uninfected control group (Figure 8C–E). Taken together, these results indicate that functional blocking with CD151 antibody or treatment with the exosome inhibitor GW4869 affects both ZIKV and DENV2 burden and reduces *cd151* expression in mosquito cells.

### 2.5. Tetraspanin CD151 Directly Interacts and Localizes with ZIKV NS2B or DENV2 Capsid or Viral Envelope Proteins in Mosquito Cells

**Figure 9:**
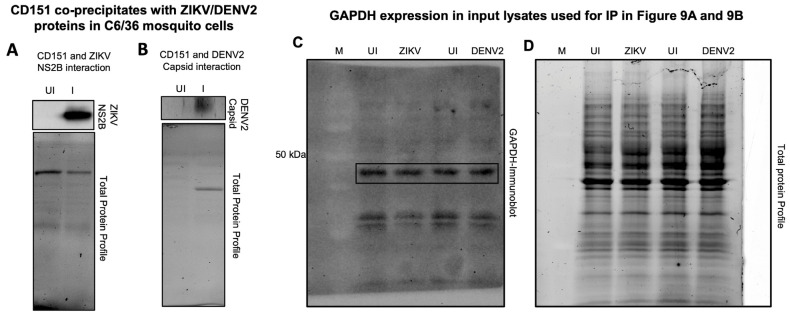
CD151 co-precipitates with ZIKV and DENV2 viral proteins in mosquito cells. Immunoprecipitation performed with uninfected (UI) and ZIKV- (**A**) or DENV2-infected (**B**) (5 MOI, for 72 h p.i.) C6/36 total cell lysates and CD151 antibody (5 µg) showing the co-precipitation of ZIKV-NS2B protein with CD151 (**A**) or DENV2 capsid protein with CD151 (**B**). Uninfected total cell lysates were used as controls in both the immunoprecipitation experiments. Total profile gel images from immunoprecipitated samples served as the input control. GAPDH expression in input lysates used for IP is shown. (**C**) Full-length immunoblot image showing GAPDH expression in input lysates used for the setting up of immunoprecipitation with the CD151 antibody and immunoblotting with ZIKV NS2B or DENV2 capsid antibodies, respectively. Immunoblot image is from chemiluminescence analysis. (**D**) Total protein profile gel images serve as the loading control. M indicates marker and the 50 kDa (kilo Dalton) band is shown.

**Figure 10:**
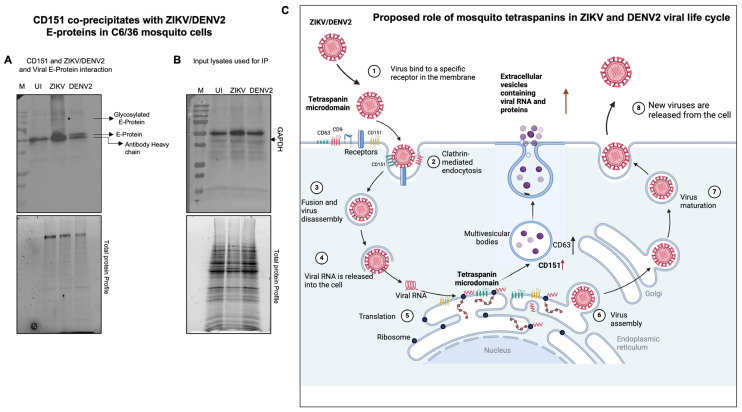
CD151 interacts with the ZIKV/DENV2 viral E-protein, and a model proposing the role of CD151 in ZIKV and DENV2 infection in mosquito cells. (**A**) Immunoblot showing direct interaction of CD151 with the ZIKV/DENV2 viral envelope (E) protein in C6/36 cells infected with respective viruses. The uninfected (UI) group serves as the control. M indicates the marker/protein ladder. The E-protein and its glycosylated forms are indicated with arrows, and CD151 antibody heavy chain is noted in all lanes (including the uninfected control group). (**B**) GAPDH levels are shown from input lysates used for immunoprecipitation assay. Total protein profile gel images (**A**,**B**) serve as controls. (**C**) The proposed model reveals CD151 could perform multiple roles in mosquitoes, including (1) serving as a specific receptor for flavivirus binding, (2) playing a potential role in clathrin-mediated endocytosis, (3) participating in fusion and virus disassembly, (4) facilitating viral RNA release, (5) enhancing the translation of viral products, (6) providing support in the virus assembly process, (7) facilitating virus maturation processes, and, (8) being an exosomal cargo molecule, CD151 may play a role in the viral exit/exocytosis process. Picture is not drawn to scale.

To characterize the role of CD151 protein in facilitating ZIKV and DENV2 infection and exosome-mediated transmission, we analyzed the direct interactions of the CD151 protein with ZIKV and DENV2 in C6/36 cells infected with 5 MOI of the respective viruses. At day 3 post-ZIKV/DENV2-infection, total cell lysates (350 µg of protein samples) processed for immunoprecipitation (IP; with a highly cross-reactive human CD151 antibody) followed by immunoblotting analysis showed the presence of ZIKV NS2B or DENV2 capsid proteins complexing with mosquito CD151 protein (Figure 9A,B). Total protein profile gel images of the immunoprecipitated lysates (used as input) served as controls (Figure 9A,B). In addition, we detected GAPDH protein levels in the input lysates (used for IP with CD151 and immunoblotting with ZIKV-NS2B or DENV2 capsid proteins), which showed no changes between the ZIKV/DENV2-infected groups and their respective uninfected loading controls (Figure 9C). Total protein profile gel images serve as loading controls (Figure 9D). The immunofluorescence analysis of CD151 protein (in green channel) and ZIKV NS2B (Figure S12A) or DENV2 capsid (Figure S12B) proteins (in red channels) revealed the co-localization of CD151 with the respective viral proteins (shown as merged or overlay images) (Figure S12A,B). Insets are shown for the clear detection and co-localization (with intense yellow fluorescence) of CD151 with ZIKV and DENV2 viral proteins (Figure S12A,B). Uninfected mosquito cell nuclei stained with DAPI along with bright field images served as controls (Figure S12A,B). To further confirm the direct interactions of CD151 protein with the ZIKV-NS2B and DENV2 capsid proteins, we selected the viral envelope (E) protein and found that CD151 interacts with both the ZIKV/DENV2 viral E-proteins (Figure 10A). Direct physical interactions of CD151 revealed an important function of this tetraspanin in facilitating ZIKV/DENV2 infection in mosquito cells. The GAPDH levels, determined from the input lysates used for immunoprecipitation are shown (Figure 10B). Overall, these data indicate that CD151 could directly interact with the ZIKV NS2B and DENV2 capsid proteins, and both viral E-proteins perhaps to facilitate the infection, transmission, and survival of these flaviviruses in mosquito cells.

## 3. Discussion

Understanding the extended and persistent infection (for several weeks) of flaviviruses in mosquito vectors requires a detailed analysis of vector–pathogen interactions. This knowledge could reveal essential details shared among diverse vectors and/or their conserved cellular factors that can be targeted to reduce flavivirus infection, transmission, dissemination, and survival. Studies like these could lead to the identification of novel therapeutic target molecules [41]. In this study, we aimed to characterize the role of mosquito transmembrane-domain-containing tetraspanin glycoproteins in relation to two medically important flaviviruses, ZIKV and DENV2, in mosquito cells. Detailed bioinformatics and prediction analysis revealed that other than Tsp29Fb (a human CD63 ortholog in mosquitoes, from our previous study), the *A. aegypti* genome harbors several tetraspanin proteins. Although mosquito tetraspanins formed different clades from one another, still they showed some degree of conservation. We tested the gene expression of several tetraspanins and identified only CD151 to be selectively and significantly upregulated in both mosquito cells (Aag-2 and C6/36 cells) and in EVs derived from these cells and upon infection with ZIKV and DENV2. These data suggest an important and persistent role for this molecule in flaviviral replication, dissemination within the vector, and transmission from vector to the mammalian host.

Two other tetraspanins (*tsps*-2 and 9) showed significant differences in gene transcripts (upregulation of *tsp*-2 at day 5 and downregulation of *tsp*-9 at day 1) post-ZIKV-infection, but their transcripts were absent in EVs, suggesting them as temporally regulated molecules. The lack of changes in the gene expression of several other tetraspanin molecules suggested that these mosquito tetraspanins have selective or non-redundant roles. One of the unique features of CD151 expression is that it is upregulated by both ZIKV and DENV2 in both mosquito cells (Aag-2 and C/36 cells) and in EVs derived from these infected cells. The presence of CD151 expression in mosquito exosomes suggested that this molecule could be a potential marker for mosquito exosomes-mediated transmission. Several tetraspanins, including the well-studied CD63, serve as exosomal markers. We believe that the expression of CD151 in EVs at later time points in ZIKV/DENV2 infection suggests a role for this tetraspanin in the exit process or exocytosis via EVs. The high degree of conservation in tetraspanin CD151 suggests similar roles in different organisms.

The observation of an increase in ZIKV/DENV2 loads that directly corelates with CD151 expression in C6/36 cells further suggested an increment-based induction of this molecule in relation to viral burden. Even though all tested mosquito tetraspanins had four transmembrane domains, the numbers of glycosylation/myristoylation and CK2 phosphorylation sites were variable between the tetraspanins. Among the glycosylation and PKC phosphorylation sites, TSP-11 did not have these two sites in comparison to other mosquito proteins. Genetic knockdown analysis for CD151 further showed reduced ZIKV and DENV2 viral burden in cells and in exosomes. Also, antibody functional blocking experiments showed reduced viral loads, suggesting the prominent role of this molecule in the replication, dissemination, and transmission of flaviviruses. The observation of reduced *cd151* transcript levels and viral burden upon GW4869 treatment further suggested a role for mosquito exosomal cargo such as CD151 in the processes of viral replication and transmission. The viral infectivity assays performed with *cd151*-dsRNA treatment, antibodies against the CD151 protein, or upon GW4869 treatment showed reduced viral infections. The reduction in viral loads upon *cd151*-dsRNA or antibody treatment directly revealed a role for CD151 in ZIKV/DENV2 viral replication and infectivity. The reduced viral loads and infectivity noted upon GW4869 treatments suggested the potential role of exosomal molecules, including CD151, and their functions related to exosome biogenesis in viral transmission or dissemination via exosomal routes. In addition, reduced ZIKV/DENV2 loads and decreased CD151 expression in treatment studies indicated a potential role for this exosomal molecule in viral transmission via EVs. Furthermore, the direct association and interaction of CD151 with ZIKV NS2B and DENV2 capsid or with both viral E-proteins suggested a functional role for this molecule in facilitating flavivirus and vector–host interactions.

As there is a growing interest in arthropod EV studies, there is increasing attention on their involvement in the pathology of vector-borne diseases. Several recent studies have highlighted the involvement of EVs in the replication and lifecycle of different viruses. As EVs are a part of natural cellular processes and the viruses depend on host cells, it may be reasoned that flaviviruses can utilize EVs for virus dissemination and transmission. However, there is still an ongoing research interest in deciphering the precise molecular mechanism by which flaviviruses exploit these pathways and their intended roles [42,43,44,45]. In our published studies, we have shown that exosomes are the potential mediators for the dissemination and transmission of arthropod-borne flaviviruses. Studies have reported increased numbers of EVs in ZIKV-infected cells compared to the numbers in uninfected cells [12]. Our findings suggested transmission of ZIKV via EVs, with potential roles for the tetraspanin glycoprotein CD63 and the SMPD3 (nSMase2, neutral sphingomyelinase 2) protein in regulating the release of infected EVs [12,46,47]. Also, it has been shown that the full-length DENV2 RNA genome and proteins/polyproteins are contained inside exosomes, suggesting that EVs are highly infectious and are capable of allowing the virus to establish infection, continue replication, and to disseminate within a vector and mammalian host [48,49]. The use of the exosome production and release inhibitor GW4869 (at a dose of 10 µM) inhibited the viral burden in mosquito cells as well as in EVs. This observation suggests that blocking EV release/secretion also reduces flavivirus infection [50]. The ubiquitous expression of CD151 in tissues and the enriched expression of tetraspanins in EVs highlights the growing influence of these molecules in our understanding of membrane dynamics and intracellular vesicular sorting [51]. Exosomes have been reported to exploit virus entry routes for the delivery of cargo. Numerous studies have indicated that tetraspanins acts as proviral factors and help to promote the entry of human cytomegalovirus, human papillomavirus (HPV), hepatitis C viruses, and mosquito-borne alphaviruses [52,53]. For instance, tetraspanin CD151 drives HPV endocytosis by regulating some integrin proteins [53]. Therefore, it is reasonable to state that exosomes are important in facilitating viral entry and dissemination and tetraspanins act as mediators for the execution of this whole process.

The co-association and co-localization of CD151 with ZIKV NS2B and DENV2 capsid proteins, as shown in immunoprecipitation and immunofluorescence analyses, suggested that this tetraspanin protein also has a role in viral replication and egress. This also confirms that CD151 can form tetraspanin-containing microdomains (TEMs) that interact with various proteins, including viral proteins in the replication site, as well as facilitating cargo sorting to allow viral proteins to be transmitted via EVs. Co-localization of viral proteins in plasma/intracellular membranes suggests the role of tetraspanins in cell-to-cell spreading/dissemination of the flaviviruses within the vector [12,51]. We proposed a model showing the important role of CD151 in ZIKV or DENV2 interactions with mosquito cells (Figure 10C). Different roles have been assumed for mosquito CD151, where it can (1) serve as a specific receptor for flavivirus binding, (2) play a potential role in clathrin-mediated endocytosis, (3) participate in fusion and virus disassembly, (4) facilitate viral RNA release, (5) enhance viral translational products, and provide support in virus assembly (6) and maturation processes. (7) We believe that CD151 may have a potential role in the viral exit/exocytosis process (8) as it is an exosomal cargo molecule. Although this study focused primarily on tetraspanin-mediated trafficking and biogenesis, the specific contributions of TSPs in ZIKV/DENV2 replication can be difficult to decipher due to complexity of the role and the functions of tetraspanins in different cellular processes. Our immediate future studies will focus on understanding the underlying molecular mechanisms of mosquito tetraspanin CD151 in flaviviral infection. In summary, the current study provides evidence that targeting arthropod CD151 affects ZIKV and DENV2 burden in both mosquito cells and in EVs derived from these infected cells. Studies like these not only provide molecular insights into the roles of TSPs such as CD151 in ZIKV- and DENV2-infected mosquito cells and in EVs derived from these cells but also aid us in the development of novel therapeutic strategies to target these and perhaps other flaviviruses.

## 4. Materials and Methods

### 4.1. Mosquito Cell Culture and Viral Infection

Both *Aedes aegypti* (Aag-2 cells) and *Aedes albopictus* (C6/36 cells) were grown at 28 °C and 5% CO_2_ in complete EMEM essential medium (obtained from ATCC-American type culture collection, Manassas, VA, USA) with 10% FBS (fetal bovine serum, VWR, Radnor, PA, USA) and 1% PSA (penicillin-streptomycin and amphotericin B, Thermo/Fisher Scientific, Inc., Waltham, MA, USA) according to the company’s guidelines. To determine the infection kinetics, ZIKV/DENV2 (PRVABC59 ZIKV strain or New Guinea C (NGC) DENV2 (serotype 2) strain, respectively) were obtained from BEI resources, Manassas, VA, USA. We plated 1 × 10^6^ Aag-2 cells or 2 × 10^5^ C6/36 mosquito cells in 12-well plates and infected with 5 MOI (multiplication of infection) of ZIKV/DENV2, respectively. After 1, 3, and 5 days, post-infection (p.i.), mosquito cells were harvested for either extraction of total cellular RNA or proteins in RLT RNA lysis buffer or in modified RIPA lysis buffer, respectively. Supernatants from each time point were collected and processed for isolation of EVs (as described in our previous publication) [12]. Pelleted EVs were also processed for RNA/protein extractions.

### 4.2. Isolation of EVs

EVs were isolated from the cell culture supernatants by following the differential ultracentrifugation method as described in our published studies [12,46]. Uninfected or ZIKV/DENV2-infected Aag-2 cells or C6/36 cells were obtained on days 1, 3, and 5 p.i. and were processed for EVs isolation. Freshly prepared EV pellets were immediately resuspended in either RNA or protein lysis buffers for extraction of total RNA/proteins, respectively, from EVs.

### 4.3. Total RNA Extraction, cDNA Synthesis, and QRT-PCR Analysis

Total RNA from Aag-2 or C6/36 mosquito cells was extracted using the Aurum Total RNA Mini kit (Bio-Rad, Hercules, CA, USA) and following the manufacturer’s instructions. Total RNA was converted to cDNA using an iScript cDNA synthesis kit (VWR, Radnor, PA, USA) and was used as a template for the amplification of viral genes, mosquito tetraspanin transcripts from Aag-2 cells, or *cd151*/*tsp-2* from C6/36 cells. QRT-PCR was performed using iQ-SYBR Green Supermix (obtained from Bio-Rad, Hercules, CA, USA/Quanta Bio, Beverly, MA (VWR, Radnor, PA, USA) or Maxima SYBR/R (Thermo/Fisher Scientific, Waltham, MA, USA) and the CFX96 Opus touch system (Bio-Rad, Hercules, CA, USA). QRT-PCR protocol for the amplification of mosquito tetraspanins is as follows, 95 °C for 3 min followed by 40 cycles of 95 °C for 10 s, 58 °C for 10 s, 72 °C for 30 s, and a final denaturation at 95 °C for 10 s. To normalize the amount of template, total RNA was quantified, and the same amount of RNA was used for gene amplifications. Standard curves were prepared using 10-fold serial dilutions starting from standard 1 to 6 of known quantities of different tetraspanin genes or ZIKV NS5 or DENV2 capsid gene fragments. The specific primers used for all mosquito tetraspanin gene fragments are shown in Supplementary Table S1. Published primers were used for ZIKV or DENV2 gene amplifications [12,54].

### 4.4. Immunoblotting Analysis

Briefly, C6/36 cells (1 × 10^6^ cells per well) were seeded in six-well plates and incubated overnight. The next day, cells were infected with 5 MOI of ZIKV/DENV2 for infection kinetics. For the *cd151* silencing experiment, Aag-2 cells were plated at a density of 5 × 10^5^ cells per well (in a 12 well plate) and infected with either 5 MOI of ZIKV or 2 MOI of DENV2. Silencing experiments were performed in Aag-2 cells with RNAi pathway. Total cell lysates were extracted in modified RIPA lysis buffer at days 1, 3 and 5 p.i. (for C6/36 cells) or at 48 h post-transfection and 24 h p.i. (for the silencing experiment with Aag-2 cells). Protein concentrations were estimated using the BCA protein assay kit (from Pierce, Thermo/Fisher Scientific, Waltham, MA, USA) and following the manufacturer’s instructions. Total protein lysates (25 μg of each sample), including uninfected or mock-dsRNA-treated controls, were loaded onto 12% SDS-PAGE gels followed by electrophoresis. Blots were blocked in 5% BSA (bovine serum albumin) buffer and probed with either ZIKV (for NS2B protein detection, Catalog Number: GTX 133308, GeneTex, Irvine, CA, USA) or for DENV2 (anti-dengue virus 2 antibody, Catalog Number: ab155042, Abcam, Waltham, MA, USA) antibodies or for CD151 (Catalog Numbers: A1930, from Abclonal, Inc. Woburn, MA, USA or sc-271216 from Santa Cruz Biotechnologies Inc., Dallas, TX, USA) followed by respective secondary antibodies (obtained from Santa Cruz Biotechnologies Inc, Dallas, TX, USA/BosterBio, Pleasanton, CA, USA). Total protein profile gel images served as loading controls. Detection was performed with a Western Bright ECL kit (from Thermo/Fisher Scientific, Waltham, MA, USA) and immunoblots were imaged using a Chemidoc imaging system (Bio-Rad, Hercules, CA, USA).

### 4.5. dsRNA Synthesis and Transfection of Aag-2 Cells

Mock-dsRNA and *cd151*-dsRNA synthesis was performed as described [12]. Briefly, the *cd151-*dsRNA fragment was amplified by using forward 5′-GCTGGCTTACCTCTACGAAAC-3′ and reverse 5′-TTGCTCGGACCGTCGCTTAG-3′ primers containing BglII and KPNI sites, respectively. The generated PCR fragment was cloned into the BglII and KPNI sites of the pL4440 double T7 script II vector, as described for studies with Tsp29Fb [12]. The selected final clone is processed for dsRNA synthesis using the MEGAscript RNAi kit (Ambion, Thermo/Fisher Scientific, Waltham, MA, USA) and by following the manufacturer’s instructions. The detailed dsRNA strategy is discussed in our previous study [12]. For dsRNA transfections, 5 × 10^5^ Aag-2 cells were seeded in 12-well plates with complete EMEM essential media containing 10% FBS. Lipofectamine reagent was used for transfection, and 750 ng of dsRNA was mixed with Lipofectamine and added to Aag-2 cells. A total of 24 h after transfection, cells were infected with 5 MOI of ZIKV or 2 MOI of DENV2. Bright field images were obtained before transfection and at 4, 24, and 48 h post-transfection or at 24 h p.i. and by using the Cytation 7 imaging system (BioTek/Agilent, Santa Clara, CA, USA). Cells were collected at 48 h post-transfection and 24 h post-ZIKV/DENV2-infection. Silencing efficiency and viral loads were determined using total RNA extractions, followed by cDNA synthesis and QRT-PCR analysis.

### 4.6. Antibody Blocking and GW4869 Inhibition Studies

Briefly, Aag-2 cells (5 × 10^5^ cells) were plated in 12-well plates and treated with 2 µg of CD151 antibody (Catalog Number: A1930; Abclonal, Inc., Woburn, MA, USA) for 4 h followed by infection with 5 MOI of ZIKV/DENV2. Treatment with isotype control antibody (Rabbit IgG, obtained from R&D Systems, Minneapolis, MN, USA) served as a control in the antibody blocking experiment. Aag-2 cells were collected at 24 h p.i. and processed for total RNA extraction followed by cDNA synthesis and QRT-PCR analysis. For inhibition with GW4869 (dihydrochloride hydrate, a pharmacological agent that blocks the production and release of EVs), Aag-2 cells (plated at density of 5 × 10^5^ cells) were treated with 10 µM of inhibitor for 4 h followed by infection (for 24 h p.i., 5 MOI) with ZIKV/DENV2. DMSO-treated cells served as mock controls in GW4869 inhibitor studies. The inhibitor-treated and ZIKV/DENV2-infected Aag-2 cells were imaged as before treatment at 4 or 24 h post-treatment. Cell lysates were collected in RLT lysis buffer for extraction of total RNA. Supernatants were collected for EV isolation and freshly isolated EVs were also resuspended in lysis buffer for extraction of total RNA followed by cDNA synthesis and QRT-PCR analysis.

### 4.7. End-Point Viral Dilution or Yield Assay

To determine the tissue culture infectious dose (TCID_50_) as virus titers for both ZIKV and DENV2, we performed several viral dilution assays, as described in the published study [12]. Briefly, Aag-2 or C6/36 mosquito cells were seeded (at densities of 2 × 10^4^ cells/well in 96 well plate and as 6–8 multiple replicates) in 225 µL of EMEM complete medium containing 10% FBS. For viral dilution assays with the mock/*cd151*-dsRNA experiment, we transfected the Aag-2 cells with 35 ng of respective dsRNA per well and incubated for 24 h (and as described in the methods above for transfection), followed by ZIKV or DENV2 infection (with various dilutions from laboratory virus stocks of 1.09 × 10^8^ pfu/mL for ZIKV and 1.06 × 10^7^ pfu/mL for DENV2, respectively). C6/36 cells treated with GW4869 inhibitor (at 10 µM, for 4 h) or mock control (with 0.1% DMSO as vehicle, treated for 4 h), or treatment of C6/36 cells with antibodies (0.3 µg/well of either isotype control or CD151) followed by infection with ZIKV or DENV2 (with laboratory viral stocks, which were serially diluted from 10^−1^ to 10^−6^), the cells were incubated for an additional 3 days. For each dilution group, at least 6–8 independent replicates were included in addition to the uninfected group that served as an internal control. Images from dilutions of 10^−2^ to 10^−4^ are shown as representative groups from mock/*cd151*-dsRNA or isotype/CD151 antibody-treated groups or from mock/GW4869 treatments. Mosquito cells were fixed using an acetone–PBS mixture (in 3:1 ratio, for 20 min at −20 °C) and plates were air dried, washed with 1 × PBS, and blocked with 5% FBS and PBS solution with 0.05% Sodium Azide for 15 min at room temperature (RT). ZIKV NS2B or DENV2 viral-Envelope (E)-protein were detected by incubation (for overnight at 4 °C) with their respective antibodies (GeneTex, Irvine, CA, USA), followed by 3 × washes with 1 × PBS. Following primary antibody incubations, cells were incubated with Alexa-488 (green)/594 (red) labeled mouse secondary antibodies, respectively (obtained from Thermo Fisher Scientific, Inc, Waltham, MA, USA), for 1 h at RT, followed by washes (3×) with 1 × PBS. Cells were then counter-stained with DAPI (0.5 µg/mL for 3 min, obtained from Thermo Fisher Scientific, Inc, Waltham, MA, USA). Plates were analyzed using the Cytation 7 imaging system (Bio-Tek/Agilent, Santa Clara, CA, USA). Images were scored for fluorescence or the presence of infection in comparison to the uninfected control (used as negative control for infection). TCID50 values are converted to pfu/mL to obtain the virus titers/yields. Representative images from dilutions of 1 × 10^−2^–1 × 10^−4^ (at 3 days p.i.) are shown. Images were obtained at 20× magnification and a scale bar of 200 µM is shown for each representative image and for the respective groups.

### 4.8. MTT Assay

To determine the viability of C6/36 cells, we seeded (in a 96-well plate) cells at a density of 2 × 10^4^ cells/well in 225 µL of EMEM complete media with 10% FBS. After overnight incubation, C6/36 cells were treated with mock (with 0.1% DMSO) or GW4869 inhibitor (at 10 µM for 4 h) followed by infection with ZIKV/DENV2 for an additional 24 h of incubation. After this incubation, we added 22.5 µL of MTT solution. The plates were wrapped (with aluminum foil) and incubated at 37 °C for another 3 h and thereafter 100 µL DMSO was added to the wells, respectively. After another incubation for 15 min at 37 °C, we determined the absorbance at optical densities of 570 nm and 690 nm. Each group had 6–8 replicates that were run as duplicates. Cell viability numbers were determined using the differences in values, with higher absorbances indicating increased viability of C6/36 cells.

### 4.9. Immunoprecipitation

Briefly, C6/36 cells (1 × 10^8^ cells) were seeded in T-25 flasks and incubated overnight, followed by infection with 5 MOI of ZIKV/DENV2 for 72 h, p.i., respectively. Cells were collected in modified RIPA lysis buffer for protein extractions. Protein concentrations were determined using a BCA (Bicinchoninic Acid Assay) kit (Pierce, Thermo/Fisher Scientific, Waltham, MA, USA). For immunoprecipitation, 350 µg of each protein sample was precleared with protein agarose A/G beads (obtained from Pierce/ThermoScientific, Inc., Waltham, MA, USA) at a ratio of 1:10 by volume and incubated at 4 °C for 45–60 min on a rotating shaker. After incubation, samples were spun at 13,000 rpm for 15 min and the supernatant was further incubated with 5 µg of CD151 antibody (Catalog Number: Ab1930; Abclonal, Inc., Woburn, MA, USA) overnight at 4 °C. Followed by CD151 antibody incubation, samples were treated with protein agarose A/G beads to capture antigen–antibody complexes. Samples were resuspended in 40 µL of SDS Laemmli buffer, boiled, chilled for 5 min, and loaded onto 12% SDS-PAGE gels. Immunoprecipitates that were resolved on gels were further processed for immunoblotting analysis (using ZIKV-NS2B or DENV2-Capsid or with highly cross-reactive 4G2 monoclonal antibody against ZIKV/DENV2 viral E-protein, followed by respective secondary antibodies) as described above [12]. For detection, ZIKV (NS2B) (GeneTex, Irvine, CA, USA) or DENV2 capsid (Abcam, Waltham, MA, USA) antibodies were used in the immunoblotting analysis and as described above.

### 4.10. Immunofluorescence Analysis

For microscopy, C6/36 cells (1 × 10^4^ cells per well) were seeded in 10-well glassy-bottom plates (Greiner Bio-one, Monroe, NC, USA). Cells were allowed to adhere overnight and then infected with 5 MOI of ZIKV/DENV2. Uninfected cells were used as control. Then, 72 h p.i, cells were fixed with 4% paraformaldehyde (at 37 °C for 20 min), permeabilized with 0.02% Triton X-100 (at RT for 10 min), washed 3 times with ice-cold 1 × PBS, and blocked with 3% BSA (at RT for 30 min). After blocking, cells were treated with a CD151 antibody (Catalog Number: Ab1930; Abclonal, Inc., Woburn, MA, USA) (in 1:250) dilution and detected with anti-rabbit Alexa 488 secondary antibody (Thermo/Fisher Scientific, Inc., Waltham, MA, USA) (in 1:2500 dilution). After three washes with 1 × PBS, cells were treated (at 4 °C for overnight) with ZIKV (for detection of NS2B protein, Genetex, Irvine, CA, USA) or DENV2 (for capsid protein detection, Abclonal, Woburn, MA, USA) (in 1:250 dilution) antibodies followed by incubation (at RT for 1 h) with Alexa 594 secondary antibodies in 1:2500 dilution (Pierce/ThermoFisher Scientific, Inc., Waltham, MA, USA). C6/36 cells were counterstained with DAPI (Pierce/ThermoFisher Scientific, Inc., Waltham, MA, USA) to visualize the nuclei. Fluorescence microscopic images were taken using the Cytation 7 imaging system (BioTek/Agilent, Santa Clara, CA, USA) at 40× magnification.

### 4.11. Statistical Data Analysis

Statistical data analysis was performed in GraphPad Prism 6 or 9 software for all the datasets. An unpaired student’s t test was used to compare the dataset with two variables, and a *p* value of less than 0.05 is considered as significant in all the analysis. For all results shown in this study, we have performed three independent experiments (with multiple biological replicates).

## Data Availability

All data in this manuscript are included in the main article file or in the Supplementary Information File. Any other additional information is available from the Corresponding author upon request.

## Supplementary Materials

The following supporting information can be downloaded at: https://www.mdpi.com/article/10.3390/ijms26157394/s1.
