# Supplementary material for: Mosquito Exosomal Tetraspanin CD151 Facilitates Flaviviral Transmission and Interacts with ZIKV and DENV2 Viral Proteins
Source: Int J Mol Sci. 2025 Jul 31;26(15):7394. doi: 10.3390/ijms26157394 (PMC12347819; doi:10.3390/ijms26157394)
Supplement: Supplementary file 1 [file ijms-26-07394-s001.zip › ijms-3643122-supplementary.pdf]

Mosquito exosomal tetraspanin CD151 facilitates flaviviral transmission and interacts with ZIKV and DENV2 viral proteins

Durga Neupane [1], Md Bayzid [1], Girish Neelakanta [1], and Hameeda Sultana [1, *]

[1] Department of Biomedical and Diagnostic Sciences, College of Veterinary Medicine, University of Tennessee, Knoxville, USA.

Running title: CD151 facilitates ZIKV/DENV2 infection in mosquito cells

Key Words: Mosquito cells, ZIKA virus, dengue virus, tetraspanins, CD151, EVs, GW4869

*** Corresponding Author**: Department of Biomedical and Diagnostic Sciences, College of Veterinary Medicine, University of Tennessee, Knoxville, TN 37996, USA

Email: hsultana@utk.edu, Phone: (865) 974-8217.

Supplementary Information File Includes Supplemental- Figures and Figure Legends.

I. **Supplemental Figures and**
II. **Supplemental Figures Legends**
III. **Supplemental Tables**
IV. **Supplemental Table Legends**

SUPPLEMENTARY FIGURES AND LEGENDs

Bioinformatic analysis of mosquito tetraspanins combed from *Aedes aegypti* genome

A

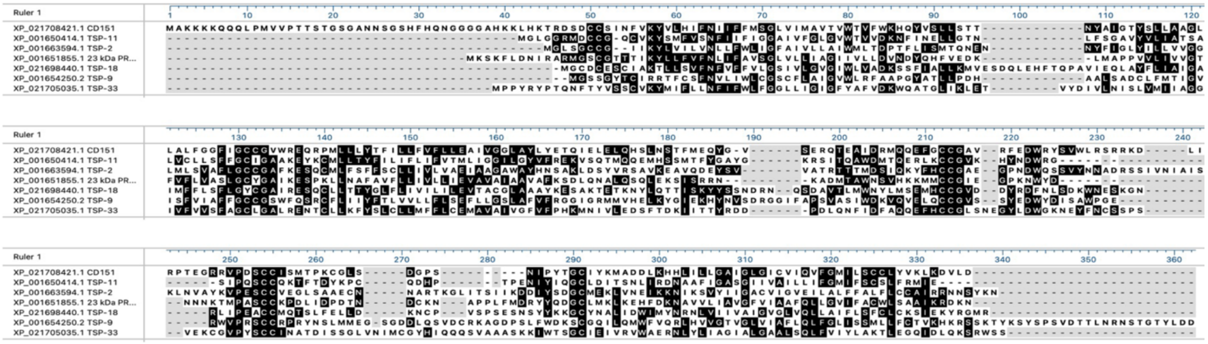

B

	XP_021708421.1 CD151	XP_001650414.1 TSP-11	XP_001663594.1 TSP-2	XP_001651855.1 23 kDa PROTEIN	XP_021698440.1 TSP-18	XP_001654250.2 TSP-9	XP_021705035.1 TSP-33
XP_021708421.1 CD151		30	26.8182	24.0909	26.3636	26.8182	27.2727
XP_001650414.1 TSP-11	0.7		25	25	28.1818	26.8182	25.9091
XP_001663594.1 TSP-2	0.731818	0.75		32.2727	26.3636	20	23.6364
XP_001651855.1 23 kDa Protein	0.759091	0.75	0.677273		27.7273	25	23.6364
XP_021698440.1 TSP-18	0.736364	0.718182	0.736364	0.722727		23.1818	25
XP_001654250.2 TSP-9	0.731818	0.731818	0.8	0.75	0.768182		25
XP_021705035.1 TSP-33	0.727273	0.740909	0.763636	0.763636	0.75	0.75	

C

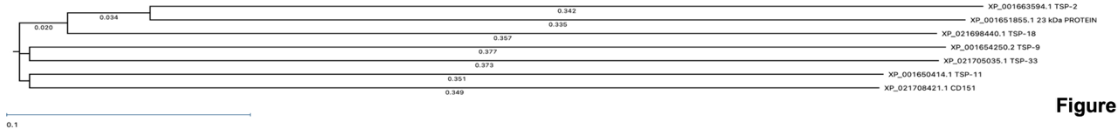

Figure S1

Figure S1. Alignment and phylogenetic analyses of *Aedes aegypti* tetraspanins combed from the genome. (A) ClustalW alignment of amino acid sequences from tetraspanin proteins combed from *A. aegypti* genome were aligned in DNASTAR laser gene software are shown. Black color represents conserved residues for easy identification. Total length of sequence is shown on top of the sequence alignment. **(B)** Table represents the percentage of identity (horizontally presented above the black box) and distance (vertically denoted below the black box) of *A. aegypti* tetraspanin proteins investigated in this study. **(C)** Phylogenetic tree analysis showing *A. aegypti* tetraspanin amino acid sequences in comparison to the other tetraspanin proteins investigated in this study. Tree was generated using the Neighbor-joining (BIONJ) method with BIONJ algorithm in DNASTAR. Phylogenetic analysis was performed using ClustalW accurate/slow alignment method in DNASTAR Lasergene. The total distance in clades is shown for each

molecule. GenBank accession numbers of each tetraspanin are provided on the left side **(A, B)** or on right side **(C)** for comparison.

Bioinformatics analysis of mosquito tetraspanin CD151 from *Aedes aegypti* genome

A

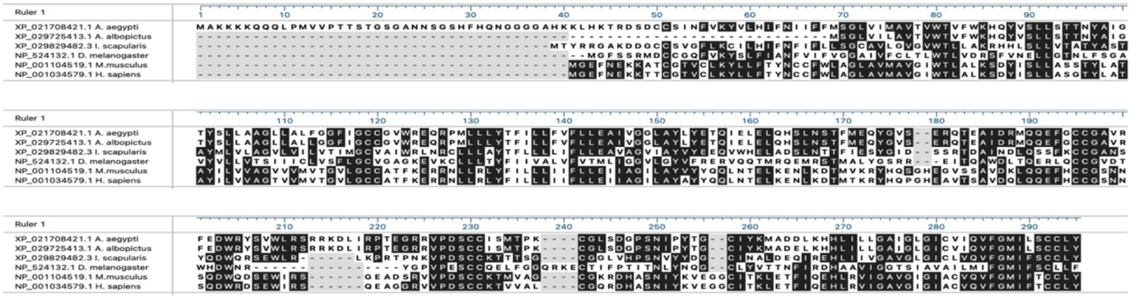

B

	XP_021708421.1 *A. aegypti*	XP_029725413.1 *A. albopictus*	XP_029829482.3 *I. scapularis*	NP_524132.1 *D. melanogaster*	NP_001104519.1 *M. musculus*	NP_001034579.1 *H. sapiens*
XP_021708421.1 *Aedes aegypti*		98.5437	48.5437	26.699	44.6602	45.1456
XP_029725413.1 *Aedes albopictus*	0.0145631		49.5146	27.1845	44.1748	44.6602
XP_029829482.3 *Ixodes scapularis*	0.514563	0.504854		29.6117	50.4854	49.0291
NP_524132.1 *Drosophila melanogaster*	0.73301	0.728155	0.703883		30.0971	29.6117
NP_001104519.1 *M. musculus*	0.553398	0.558252	0.495146	0.699029		93.6893
NP_001034579.1 *H. sapiens*	0.548544	0.553398	0.509709	0.703883	0.0631068	

C

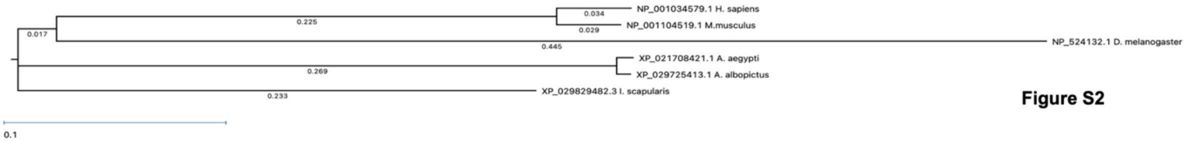

Figure S2

Figure S2. Alignment and phylogenetic analyses of *A. aegypti* CD151 tetraspanin with other orthologs. *Aedes aegypti* CD151 amino acid sequence alignment **(A)** with CD151 from orthologs (*A. albopictus*, *I. scapularis*, *D. melanogaster*, *M. musculus*, *H. sapiens*) using the ClustalW alignment in DNASTAR laser gene software are shown. Residues that are conserved are shaded in black color for easy identification. **(B)** Table showing percent identity (horizontally presented above the black box) and distance (vertically denoted below the black box) analyzed from the ClustalW alignment of *A. aegypti* CD151 tetraspanin proteins amino acid sequence to the other orthologs. **(C)** Phylogenetic tree analysis showing *A. aegypti* CD151 amino acid sequences in comparison to CD151 proteins from other orthologs. Tree was generated using the Neighbor- joining (BIONJ) method with BIONJ algorithm in DNASTAR. Phylogenetic analysis was performed using ClustalW accurate/slow alignment method in DNASTAR Lasergene. Total distance in clades is shown for each molecule. GenBank accession numbers of each CD151 is provided on the left side **(A, B)** or on right side **(C)** for comparison.

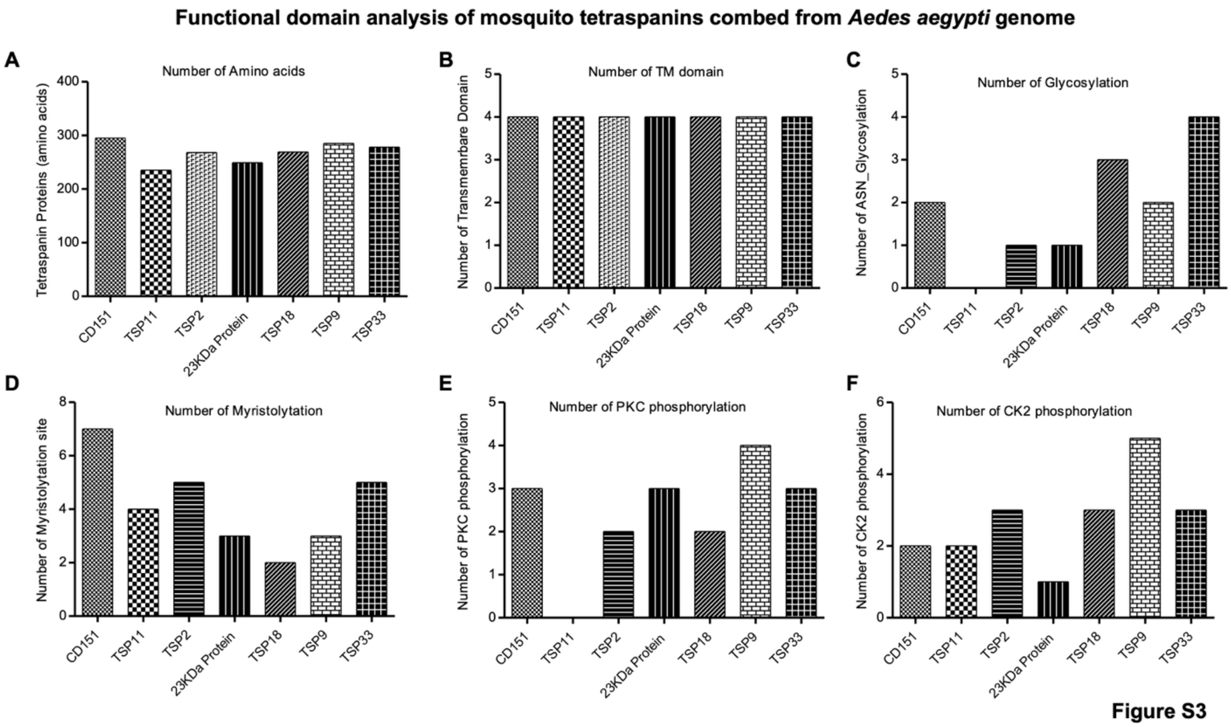

Figure S3. Functional domain and prediction analyses of mosquito tetraspanins combed from *A. aegypti* genome. Domain analysis and post-translational modification prediction showing number of amino acids (Y-axis) with corresponding accession numbers (X-axis) present in each of the mosquito tetraspanin **(A)**, number of tetraspanin domains contained in each of the mosquito tetraspanins combed from *A. aegypti* genome analyzed using TMHMM server v.2.0 **(B)**, or number of ASN glycosylation sites **(C)**, or number of Myristylations sites **(D)**, or number of PKC phosphorylation sites **(E)**, and lastly the number of CK2 phosphorylation sites **(F)**.

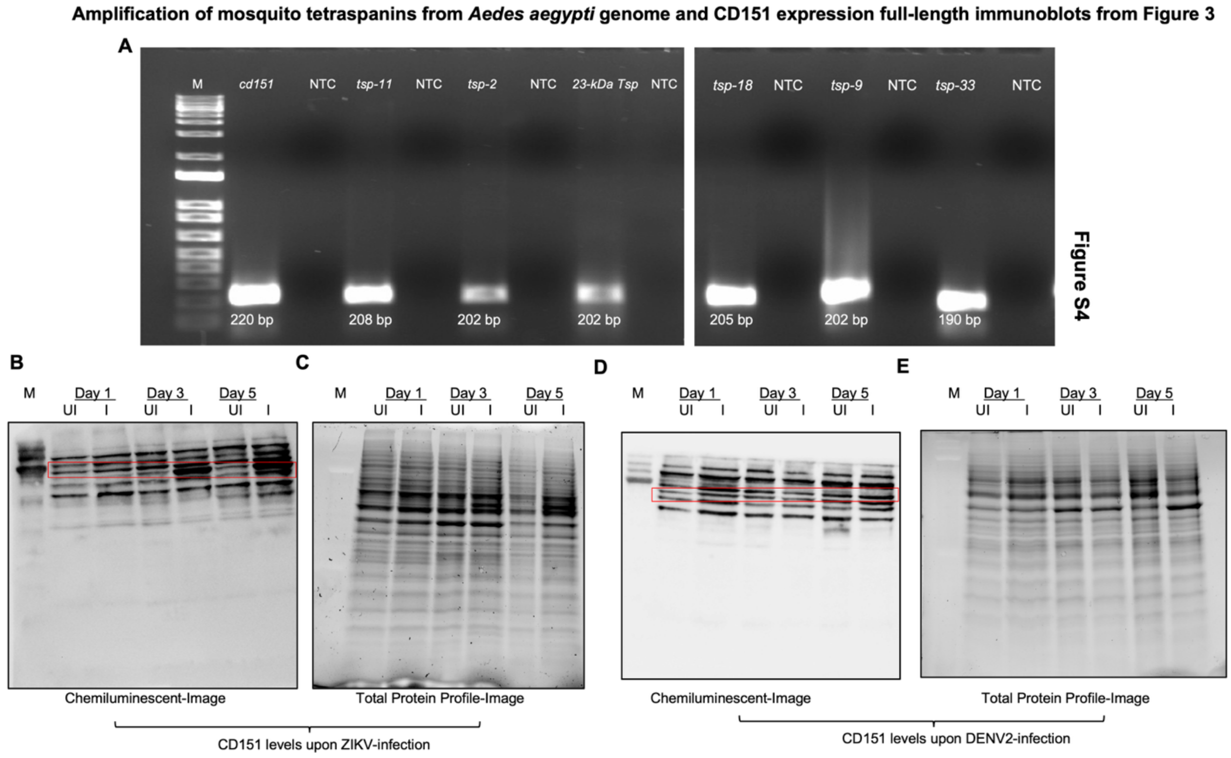

Figure S4. Amplification of mosquito tetraspanins from *A. aegypti* genome and CD151 expression full-length immunoblots from Figure 3. Agarose gel electrophoresis images **(A)** showing the amplified gene fragments for *cd151* (220 bp), *tsp-11* (208 bp), *tsp-2* (202 bp), *23-kDa protein* (202 bp), *tsp-18* (205 bp), *tsp-9* (202 bp) and *tsp-33* (190 bp) used for generation of standards used in the QRT-PCR analysis. M indicates marker/DNA ladder, NTC denotes no template control and bp represents base pairs. **(B-D)** Full-length immunoblots and total protein profile images (from Figure 3G and H) shown for CD151 expression upon ZIKV **(A, B)** and DENV2 **(C, D)** infection. Immunoblot images are from chemiluminescence, and total protein profile gel images are the TCE stained SDS-PAGE gel images.

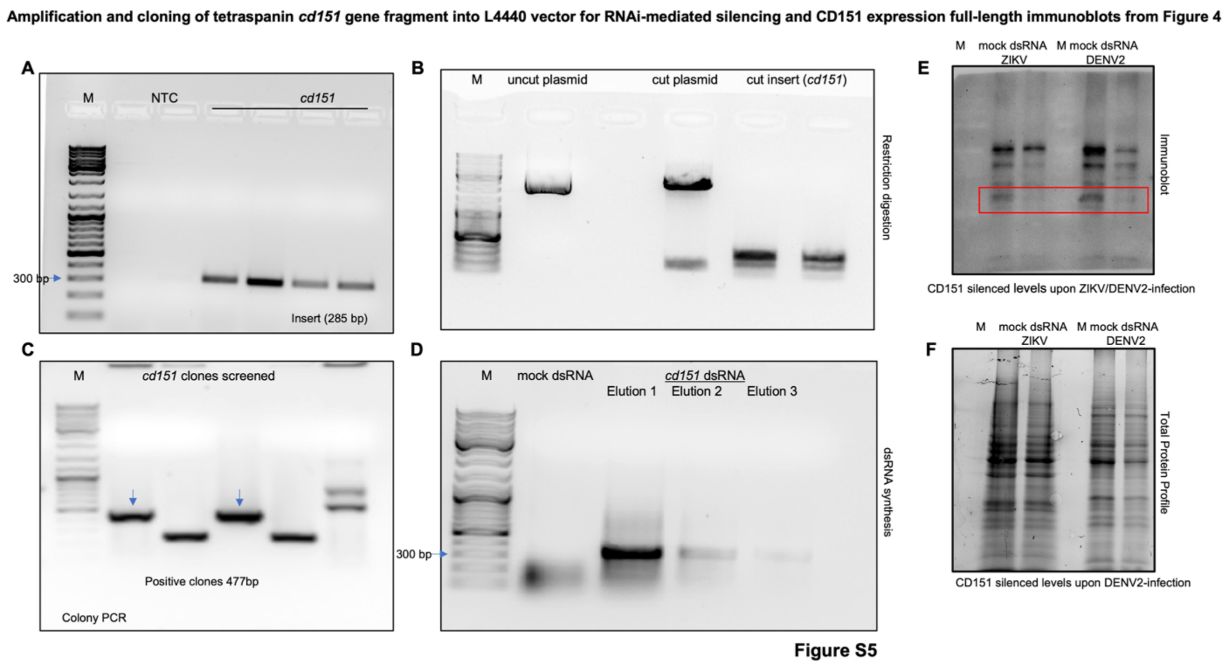

Figure S5. Amplification and cloning of tetraspanin *cd151* gene fragment into pL4440 vector for RNAi-mediated silencing and CD151 expression full-length immunoblots from Figure 4. (A) Agarose gel electrophoresis images showing the amplified gene fragments for *cd151* (285 bp). **(B)** pL4440 uncut plasmid, cut plasmid digested with BglII and KpnI enzymes to release the insert or *cd151* insert cut with enzymes. **(C)** Clones screened for presence of *cd151* gene fragment into pL4440 (447 bp) plasmid are shown. **(D)** Agarose gel images showing mock-dsRNA and three elution's from *cd151*-dsRNA. M indicates marker/DNA ladder, NTC denotes no template control and bp represents base pairs. **(E)** Full-length immunoblot image (from Figures 4I and J) shown for CD151 expression upon ZIKV and DENV2 infection. Immunoblot image is from chemiluminescence. **(F)** Total protein profile gel image serve as loading control.

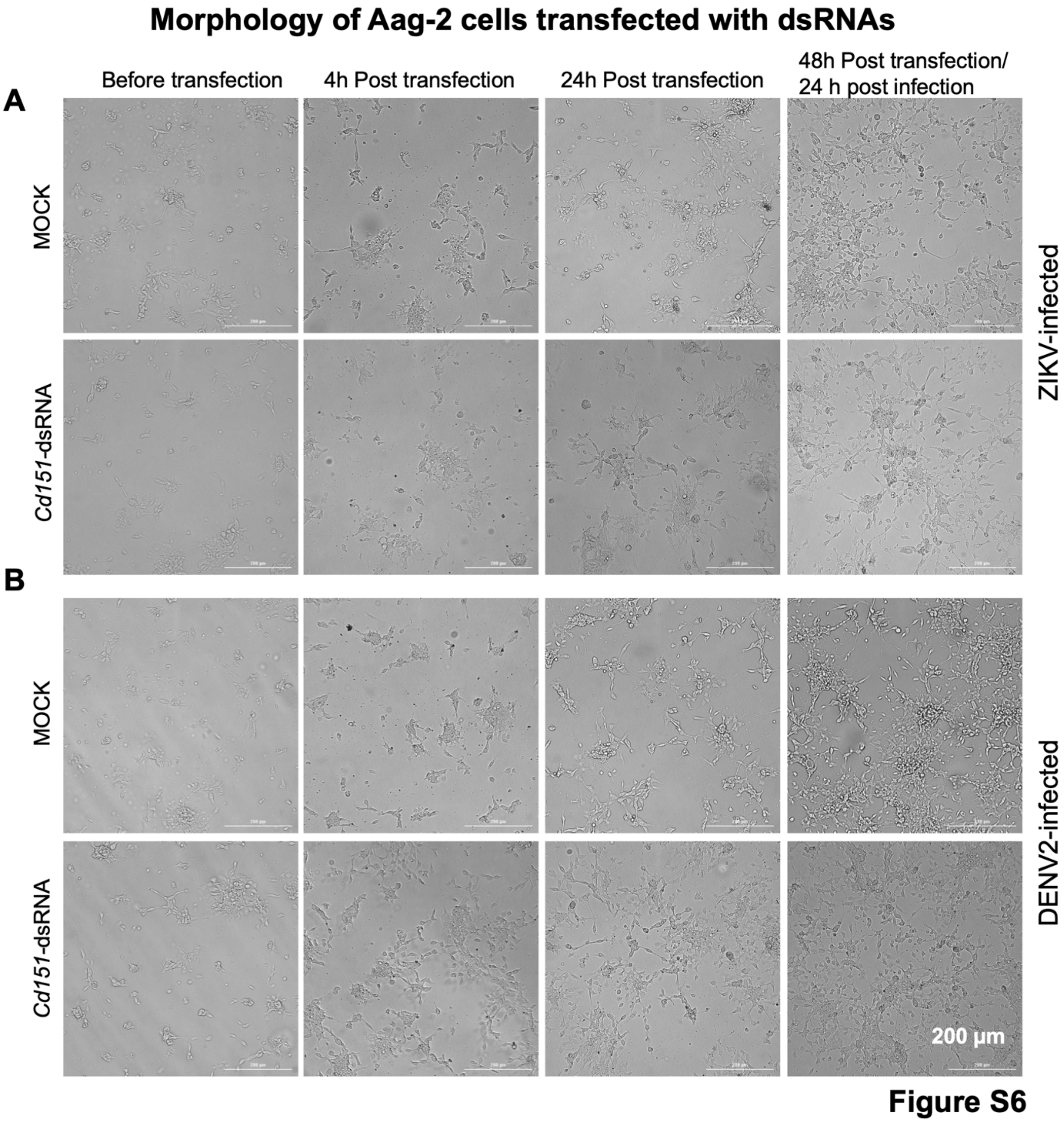

Figure S6. **Morphology of *A. aegypti* cells transfected with *cd151* dsRNA.** Phase contrast microscopic images of ZIKV (5 MOI) **(A)** or DENV2 (2 MOI)-infected **(B)** Aag-2 cells treated with mock-dsRNA (empty pL4440 plasmid), or *cd151*-dsRNA are shown from before transfection, followed by 4 h, 24 h and 48 h post transfection or 48 h post infection. The mock-dsRNA-treated groups with either ZIKV-infection **(A)** or DENV2-infection **(B)** are shown as

respective controls. All images were obtained using Cytation 7 imaging system and scale bar indicates 200 μm.

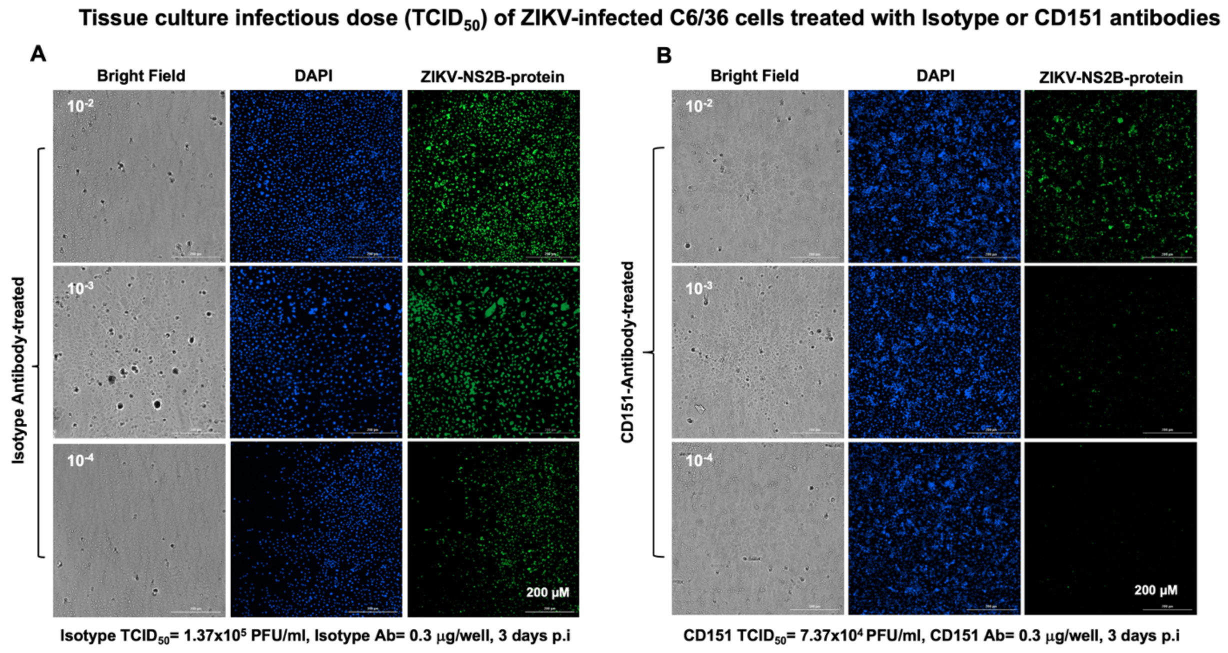

Figure S7

Figure S7. ZIKV infectivity is reduced upon antibody blocking for CD151 in C6/36 cells. Viral dilution or infectivity assay showing tissue culture infectious dose ($TCID_{50}$) that determines the viral infectivity (by immunofluorescence assay) in isotype antibody-treated **(A),** or CD151 antibody-treated **(B)** C6/36 cells infected with ZIKV (for 3 days p.i.) at different dilutions of 10^{-2}, 10^{-3} and 10^{-4}. Bright field images of C6/36 cells from ZIKV-infected groups are shown on left column. DAPI stained nuclei images are shown in blue color (and in the middle). ZIKV-NS2B viral protein staining is shown in green color (on the right). The $TCID_{50}$ dose is indicated at the bottom of each panel. Images are obtained at 20X magnification. Scale bar indicates 200 μm in each image and highlighted in the last image of the panel.

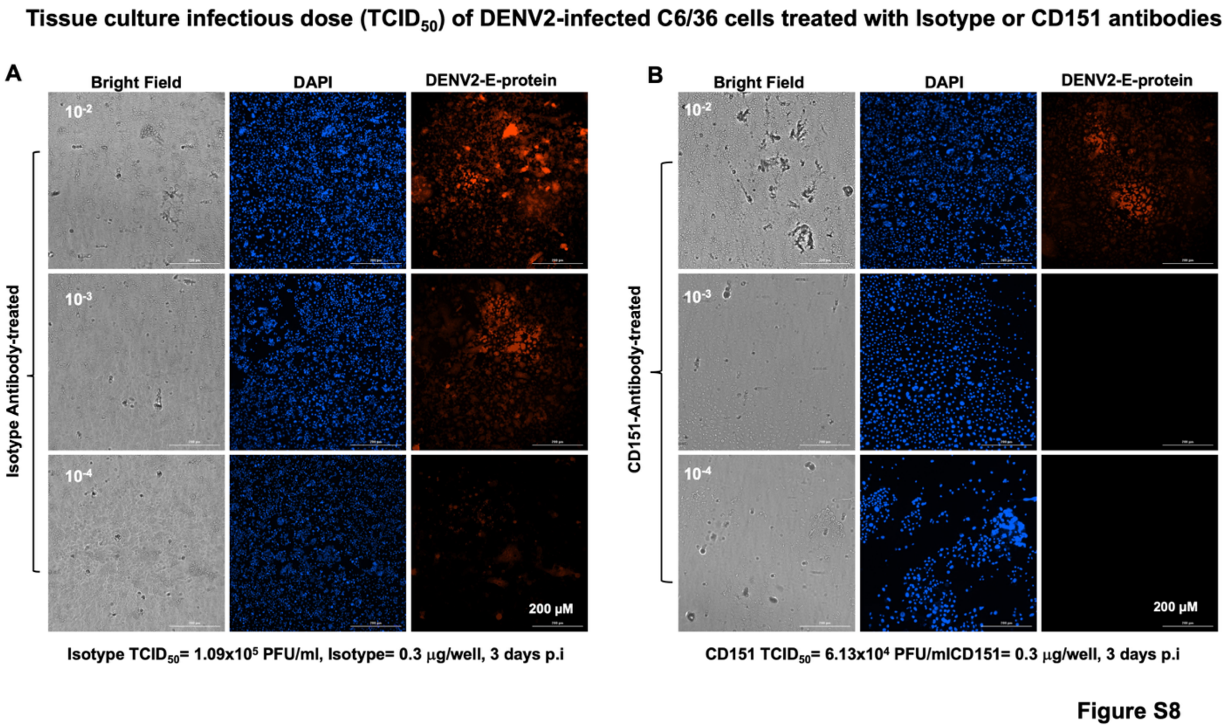

Figure S8. DENV2 infectivity is reduced upon antibody blocking for CD151 in C6/36 cells. Viral dilution or infectivity assay showing tissue culture infectious dose ($TCID_{50}$) that determines the viral infectivity (by immunofluorescence assay) in isotype antibody-treated **(A),** or CD151 antibody-treated **(B)** C6/36 cells infected with DENV2 (for 3 days p.i.) at different dilutions of 10^{-2}, 10^{-3} and 10^{-4}. Bright field images of C6/36 cells from DENV2-infected groups are shown on left column. DAPI stained nuclei images are shown in blue color (and as middle column). DENV2-capsid viral protein staining is shown in green color (and on the right column). The $TCID_{50}$ dose is indicated at the bottom of each panel. Images are obtained at 20X magnification. Scale bar indicates 200 µm in each image and highlighted in the last image of the panel.

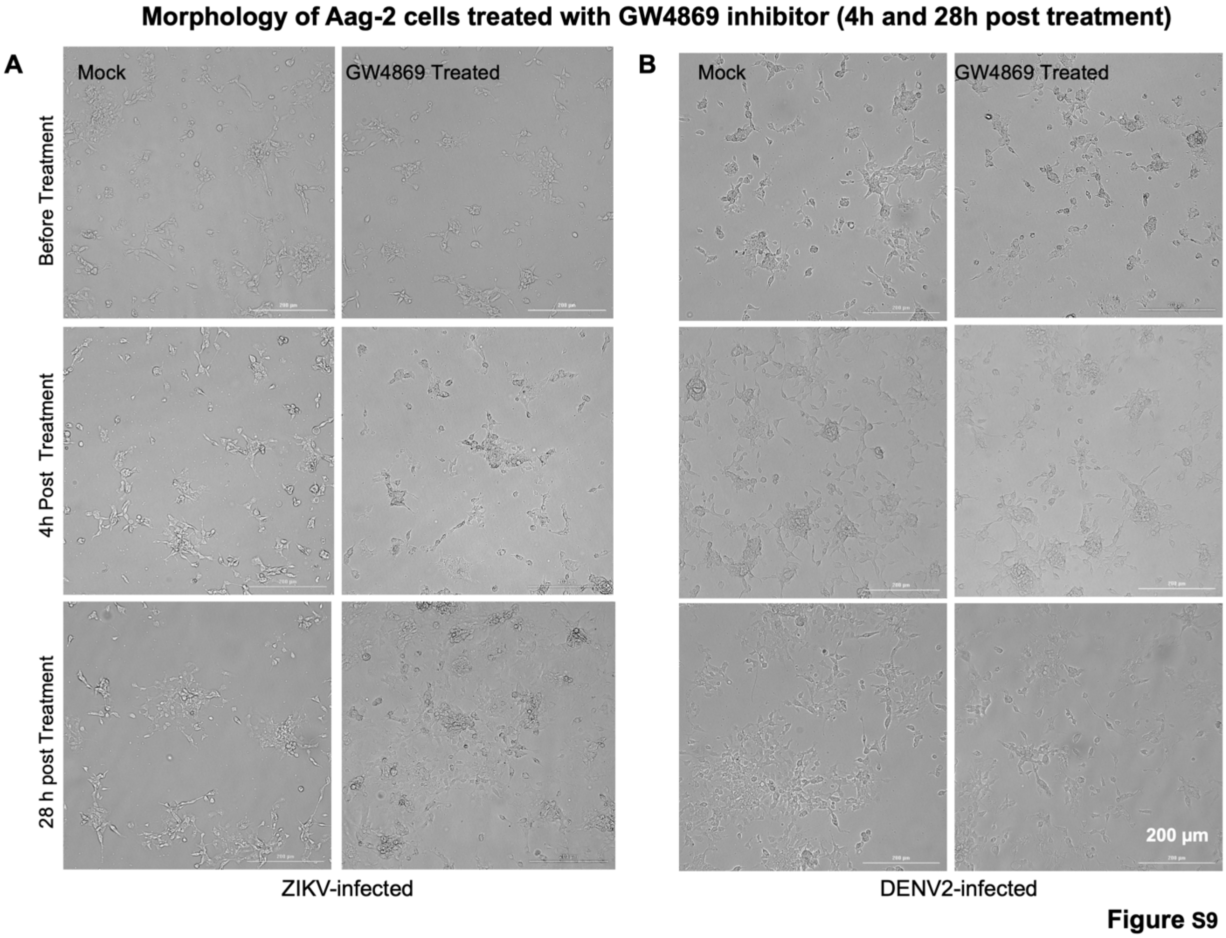

Figure S9. Morphology of *A. aegypti* cells treated with GW4869 inhibitor. Phase contrast images of mock (DMSO 0.1%) or GW4869-treated (at 10 µM, for 4 h) Aag-2 cells infected with either ZIKV **(A)** or DENV2 **(B)** (with 5 MOI for 24 h) are shown. The mock-treated groups with either ZIKV **(A)** or DENV2-infection **(B)** are shown as respective controls. All images were obtained using Cytation 7 imaging system and scale bar indicates 200 µm.

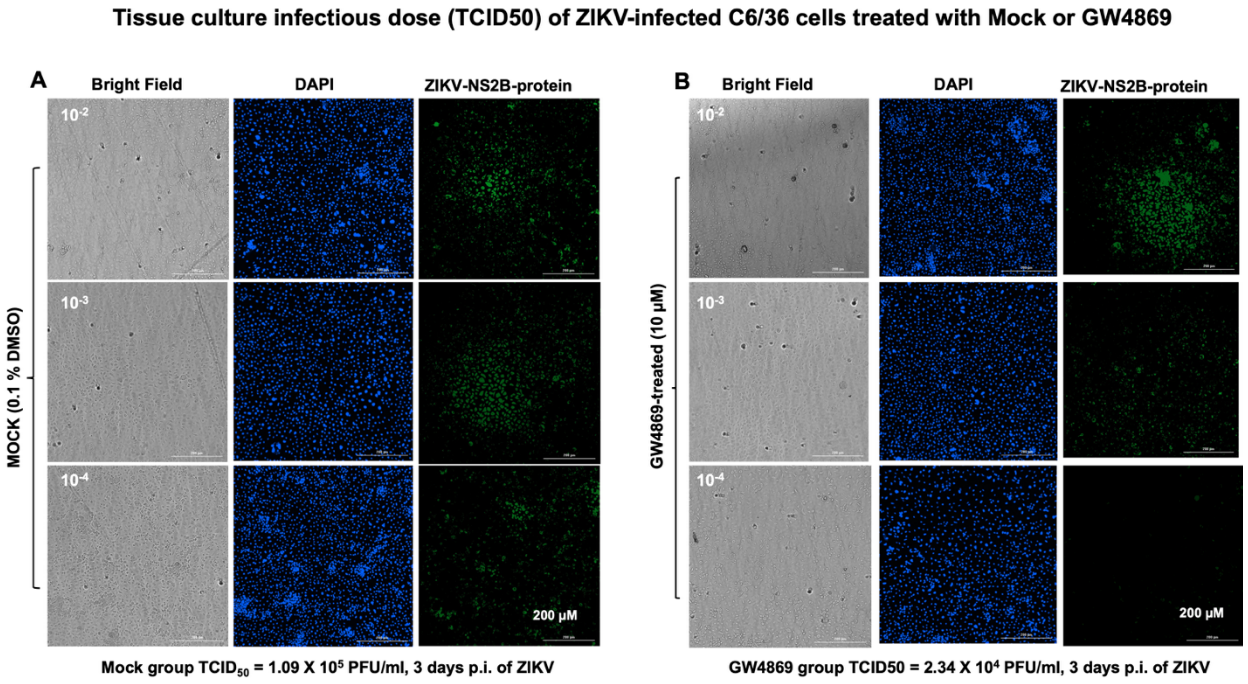

Figure S10

Figure S10. ZIKV infectivity is reduced upon GW4869 treatment in C6/36 cells. Viral dilution or infectivity assay showing tissue culture infectious dose ($TCID_{50}$) that determines the viral infectivity (by immunofluorescence assay) in mock-treated (0.1% DMSO, for 4 h) **(A),** or GW4868-treated (at 10 μM, for 4 h) **(B)** C6/36 cells infected with ZIKV (for 3 days p.i.) at different dilutions of 10^{-2}, 10^{-3} and 10^{-4}. Bright field images of C6/36 cells from ZIKV-infected groups are shown on left column. DAPI stained nuclei images are shown in blue color (and in the middle). ZIKV-NS2B viral protein staining is shown in green color (on the right). The $TCID_{50}$ dose is indicated at the bottom of each panel. Images are obtained at 20X magnification. Scale bar indicates 200 μm in each image and highlighted in the last image of the panel.

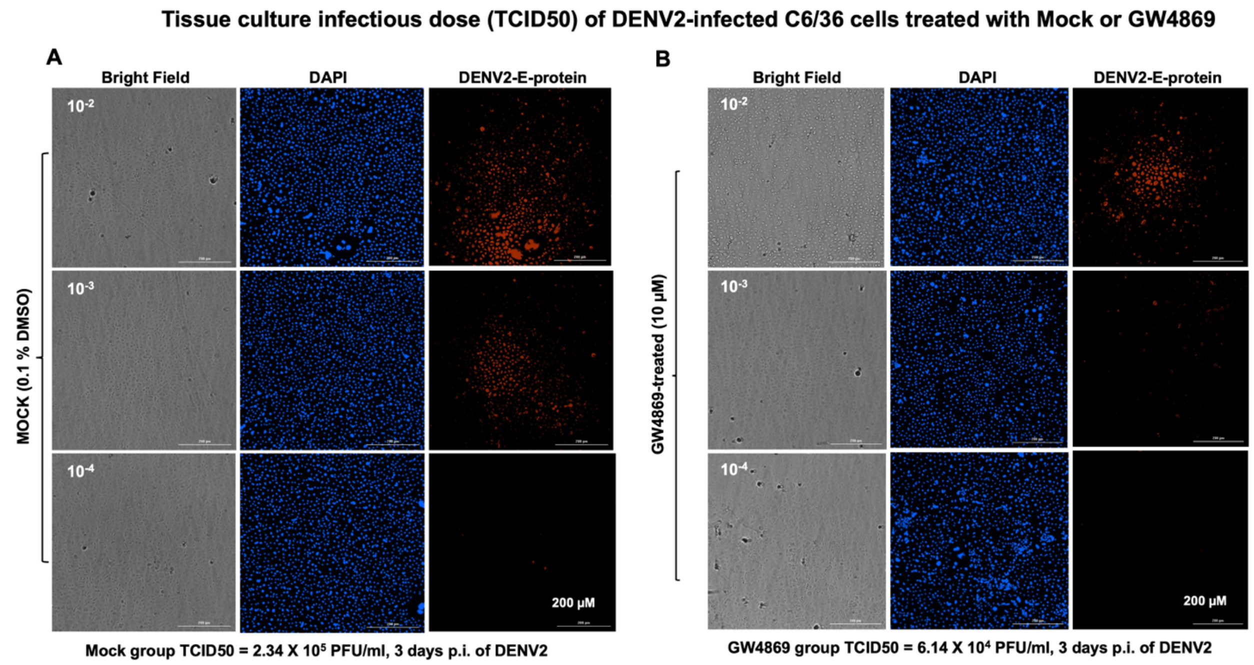

Figure S11

Figure S11. DENV2 infectivity is reduced upon GW4869 treatment in C6/36 cells. Viral dilution or infectivity assay showing tissue culture infectious dose ($TCID_{50}$) that determines the viral infectivity (by immunofluorescence assay) in mock-treated (0.1% DMSO, for 4 h) **(A),** or GW4868-treated (at 10 µM, for 4 h) **(B)** C6/36 cells infected with DENV2 (for 3 days p.i.) at different dilutions of 10^{-2}, 10^{-3} and 10^{-4}. Bright field images of C6/36 cells from DENV2-infected groups are shown on left column. DAPI stained nuclei images are shown in blue color (and in the middle). DENV2-capsid viral protein staining is shown in green color (on the right). The $TCID_{50}$ dose is indicated at the bottom of each panel. Images are obtained at 20X magnification. Scale bar indicates 200 µm in each image and highlighted in the last image of the panel.

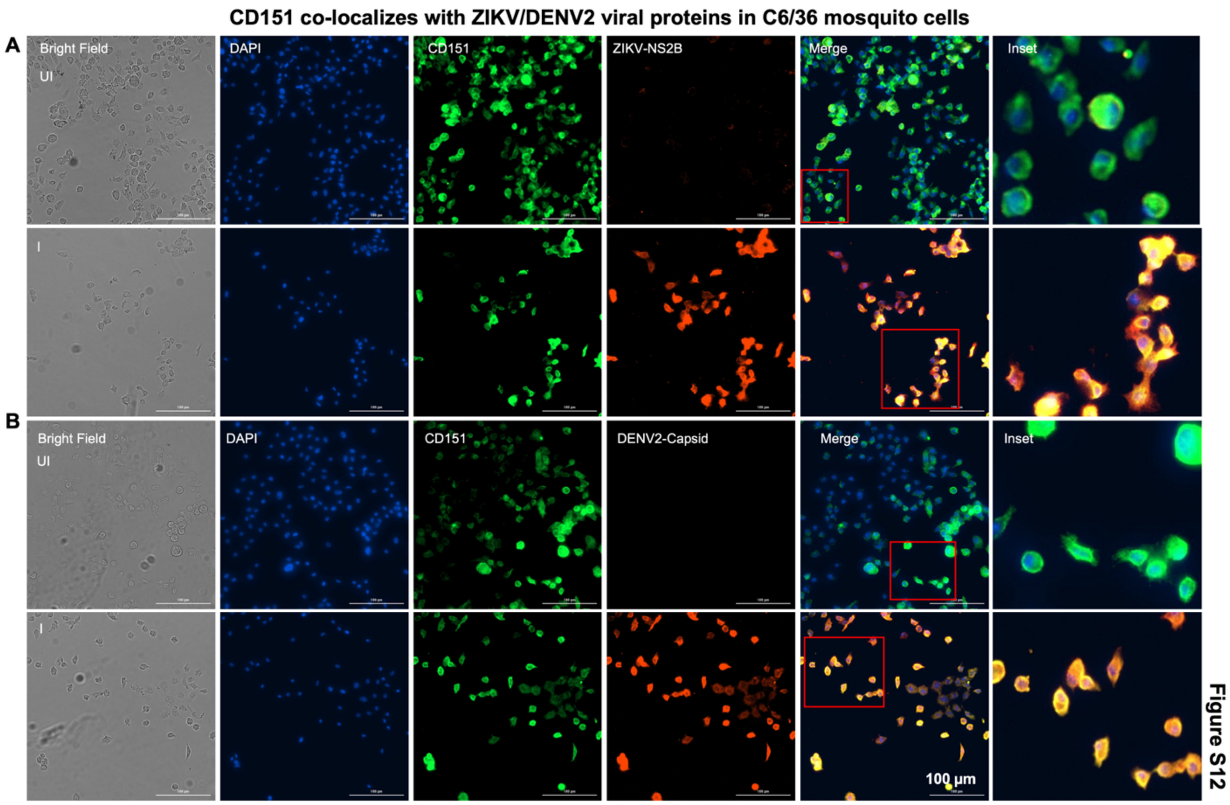

Figure S12. Microscopic analysis for distribution of CD151 and viral proteins.

Immunofluorescence analysis showing the co-localization of ZIKV (NS2B) **(A)** or DENV2 (capsid) proteins **(B)** with CD151 protein in infected (5 MOI, for 72 h p.i.) C6/36 mosquito cells. Bright field images of C6/36 cells from uninfected or ZIKV or DENV2-infected groups are shown. DAPI stained nuclei images are shown in blue color. CD151 protein staining is shown in green color, ZIKV NS2B or DENV capsid proteins staining is shown in red color. Yellow color in merged images indicates co-localization. Yellow color in merged images indicates co-localization. Insets (red boxed sections) from merged images are shown in column 6. Scale bar indicates 100 µm in each image.

SUPPLEMENTARY TABLES AND LEGENDS

Mosquito Tetraspanins combed from *Aedes aegypti* genome

Type	Name	Protein accession	NCBI nucleotide Accession	VectorBase Accession	Signal peptide
1	CD151	XP_021708421.1	XM_021852729.1	AAEL005142	No
2	Tetraspanin 11	XP_001650414.1	XM_001650364.2	AAEL005137	No
3	Tetraspanin 2 Isoform	XP_001663594.1	XM_001663544.2	AAEL013404	No
4	23 kDa Integral membrane protein	XP_001651855.1	XM_001651805.2	AAEL006282	No
5	Tetraspanin 18	XP_021698440.1	XM_021842748.1	AAEL014311	No
6	Tetraspanin 9	XP_001654250.2	XM_001654200.2	AAEL010102	No
7	Tetraspanin 33	XP_021705035.1	XM_021849343.1	AAEL004147	No

Table S1

Table S1. Mosquito tetraspanin proteins combed from *A. aegypti* genome. Table 1 showing the names of tetraspanins, their protein and nucleotide NCBI accession numbers, VectorBase accession numbers and signal peptides presence/absence are shown. The tetraspanin protein name, protein and nucleotide accession numbers were collected from NCBI. The presence/absence of signal peptide was determined at TMHMM website.

Oligonucleotides used in this study

Primers(5'-3')	Purpose
F-5' TCGGTGGATTCATCGGATGC-3'<br>R-5' CGATCGATGGCTTCGGTTTG-3'	*Cd151* (220 bp fragment), QPCR
F-5'CGTGACGATGCTCATCGGA3'<br>R-5'AGACTGTGGGATCGAACCG3'	*tsp-11* (208 bp fragment), QPCR
F-5' GCTGCCTTCTGATCGTTCT-3'<br>R-5'CAATCATTAGGACCCTCCG-3'	*tsp-2* (202 bp fragment), QPCR
F-5' GCAGAATGCTCTGCAATCGC-3'<br>F-5' CTTGCAGTCGTTGGTATCGG-3'	23 kDa integral membrane protein (202 bp fragment), QPCR
F-5' GCGATACGAGAAAGCCAGT-3'<br>F-5' TCCACATCAACGTGACAGC-3'	*tsp-18* (205 bp fragment), QPCR
F-5' GCTGTTCCTGAGCGAGTTTC-3'<br>F-5' GTACGAACTGACTCCACAGC-3'	*tsp-9* (202 bp fragment), QPCR
F-5' GCTGATGTTCTTCCTGTGCG-3'<br>F-5' GCCTTCGTTGCTCAATCCAC-3'	*tsp-33* (190 bp fragment),QPCR
F-5' TGAGATCTGCTGGCTTACCTCTACGAAAC -3'<br>R-5' CGGGTACCTTGCTCGGACCGTCGCTTAG -3'	*Cd151* (285 bp fragment), RNAi Analysis
F-5' AATATGCTGAAACGCGAGAGAAACCGCG-3'<br>R-5'CTCTTCAGTATCCCTGCTGTTGG-3'	DENV2, capsid
F-5' CCTTGGATTCTTGAACGAGGA-3'<br>R-5' AGAGCTTCATTCTCCAGATCAA-3'	ZIKV, NS5

Table S2

Table S2. Oligonucleotides used in this study. Table 2 showing the sequences of oligonucleotides used to amplify the mosquito tetraspanin gene fragments. Fragment sizes (in base pairs; bp) are indicated in the right column with respective gene label. The oligonucleotide sequences are shown from 5'-3'.
